# Superpixel-ComBat modeling: A joint approach for harmonization and characterization of inter-scanner variability in T1-weighted images

**DOI:** 10.1162/imag_a_00306

**Published:** 2024-10-03

**Authors:** Chang-Le Chen, Mahbaneh Eshaghzadeh Torbati, Davneet S. Minhas, Charles M. Laymon, Seong Jae Hwang, Murat Bilgel, Adina Crainiceanu, Hecheng Jin, Weiquan Luo, Pauline Maillard, Evan Fletcher, Ciprian M. Crainiceanu, Charles S. DeCarli, Howard J. Aizenstein, Dana L. Tudorascu

**Affiliations:** Department of Bioengineering, University of Pittsburgh, Pittsburgh, PA, United States; Intelligent System Program, School of Computing and Information, University of Pittsburgh, Pittsburgh, PA, United States; Department of Radiology, School of Medicine, University of Pittsburgh, Pittsburgh, PA, United States; Department of Artificial Intelligence, Yonsei University, Seoul, South Korea; Laboratory of Behavioral Neuroscience, National Institute on Aging, National Institutes of Health, Baltimore, MD, United States; Computer Science Department, United States Naval Academy, Annapolis, MD, United States; Department of Neurology, University of California Davis, Davis, CA, United States; Department of Biostatistics, Johns Hopkins University, Baltimore, MD, United States; Department of Psychiatry, School of Medicine, University of Pittsburgh, Pittsburgh, PA, United States; Department of Biostatistics, University of Pittsburgh, Pittsburgh, PA, United States

**Keywords:** inter-scanner variability, T1-weighted imaging, ComBat, harmonization, superpixel

## Abstract

T1-weighted imaging holds wide applications in clinical and research settings; however, the challenge of inter-scanner variability arises when combining data across scanners, which impedes multi-site research. To address this, post-acquisition harmonization methods such as statistical or deep learning approaches have been proposed to unify cross-scanner images. Nevertheless, how inter-scanner variability manifests in images and derived measures, and how to harmonize it in an interpretable manner, remains underexplored. To broaden our knowledge of inter-scanner variability and leverage it to develop a new harmonization strategy, we devised a pipeline to assess the interpretable inter-scanner variability in matched T1-weighted images across four 3T MRI scanners. The pipeline incorporates ComBat modeling with 3D superpixel parcellation algorithm (namely SP-ComBat), which estimates location and scale effects to quantify the shift and spread in relative signal distributions, respectively, concerning brain tissues in the image domain. The estimated parametric maps revealed significant contrast deviations compared to the joint signal distribution across scanners (*p*< 0.001), and the identified deviations in signal intensities may relate to differences in the inversion time acquisition parameter. To reduce the inter-scanner variability, we implemented a harmonization strategy involving proper image preprocessing and site effect removal by ComBat-derived parameters, achieving substantial improvement in image quality and significant reduction in variation of volumetric measures of brain tissues (*p*< 0.001). We also applied SP-ComBat to evaluate and characterize the performance of various image harmonization techniques, demonstrating a new way to assess image harmonization. In addition, we reported various metrics of T1-weighted images to quantify the impact of inter-scanner variation, including signal-to-noise ratio, contrast-to-noise ratio, signal inhomogeneity index, and structural similarity index. This study demonstrates a pipeline that extends the implementation of statistical ComBat method to the image domain in a practical manner for characterizing and harmonizing the inter-scanner variability in T1-weighted images, providing further insight for the studies focusing on the development of image harmonization methodologies and their applications.

## 1 Introduction

T1-weighted imaging, as one of the most widely employed structural magnetic resonance imaging (MRI) techniques, plays a pivotal role in clinical and research settings for diagnostic purposes and provides valuable neuroanatomical information (Bethlehem et al., 2022;C.-L. Chen, Kuo, Wu, et al., 2022;Jalbrzikowski et al., 2021;Mattsson et al., 2019). To enhance statistical power and generalizability of research outcomes, there is a growing interest in aggregating structural MRI data from multiple sites (Bethlehem et al., 2022;Nemoto et al., 2020). However, inter-scanner variability poses a challenge in multicenter studies (Chen et al., 2020;Pomponio et al., 2020;Torbati et al., 2021), arising from differences in imaging acquisition across scanners (Han et al., 2006;Hedges et al., 2022;Takao et al., 2014). Technical factors such as hardware design, field strengths, field inhomogeneity, imaging protocols, and software upgrades may contribute to variations in image quality factors such as noise, contrast, and signal inhomogeneity (Han et al., 2006;Hedges et al., 2022;Jovicich et al., 2006;Takao et al., 2014). This variability can affect the segmentation of primary tissue types, including gray matter (GM), white matter (WM), and cerebrospinal fluid (CSF), influencing downstream analyses and lowering accuracy and reproducibility of biomarkers derived from structural MRI data (Chen et al., 2024;Han et al., 2006;Liu et al., 2020;Srinivasan et al., 2020;Takao et al., 2014). Although standardization efforts at image processing can partially mitigate these effects, changes in acquisition settings still introduce significant variability (Gebre et al., 2023). Consequently, post-acquisition harmonization methods are crucial for ensuring the reliability and consistency of biomarkers in disease diagnosis and prognosis across different scanners (Hu et al., 2023). These methods help address the challenges posed by inter-scanner variability and promote accurate and reliable quantitative measurements in both cross-sectional and longitudinal studies (Gebre et al., 2023;Richter et al., 2022).

To address the challenge of inter-scanner variability in T1-weighted images, several retrospective harmonization approaches have been proposed, targeting different levels of data formation and subsequent analytic procedures (e.g., image level or feature level) (Dewey et al., 2019;Pomponio et al., 2020;Torbati et al., 2021). Harmonization involves adjusting the domain-specific characteristics of the source and/or target scans to ensure consistency between them (Hu et al., 2023;Marzi et al., 2024). The general goal of harmonization is to achieve indistinguishable image-related characteristics such as contrast, texture, and feature distribution between scanners (Hu et al., 2023). In practice, image translation techniques such as histogram matching, intensity warping, and deep learning-based methods are commonly employed to harmonize source scans by learning the mapping between source and target images at the image level (Dewey et al., 2019;Shinohara et al., 2014;Torbati et al., 2023;Wrobel et al., 2020;Zuo et al., 2023). Another category of harmonization methods focuses on removing scanner-related effects by utilizing statistics-based techniques to analyze parameters of distributions at the feature level (Beer et al., 2020;A. A. Chen et al., 2022;Radua et al., 2020;Zhang et al., 2023). Both categories offer harmonization methods implemented either in a matched manner (i.e., involving traveling subjects) or in an unmatched manner (i.e., without the need for traveling subjects) (Hu et al., 2023). In the statistics-based approaches, ComBat and its variants have gained popularity for harmonizing brain imaging (Beer et al., 2020;A. A. Chen et al., 2022;Fortin et al., 2018). ComBat is a batch harmonization technique for tabular data that employs empirical Bayes estimation to approximate the scanner effect (Richter et al., 2022;Torbati et al., 2021). The method models cross-scanner data as a linear combination of variables of interest, covariates, and scanner effects that are estimated by additive and multiplicative terms to characterize the location (i.e., mean) and scale (i.e., variance) effects of feature distributions (Johnson et al., 2007). ComBat assumes that scanner effects come from a joint distribution that can be modeled by a point estimation of latent variables (i.e., pooling scanner effects towards a mean) through the empirical Bayes estimation, making the modeling more robust and computationally less expensive (Johnson et al., 2007;Reynolds et al., 2023).

The current literature on neuroimaging harmonization indicates that existing approaches demonstrate divergent effectiveness depending on specific conditions (e.g., the types of image modality) and the intended purposes (e.g., harmonization at the feature distribution level or image quality) (Hu et al., 2023); however, it reveals several gaps in understanding how image quality varies across scanners and interpreting how harmonization methods handle this discrepancy. Recently developed deep learning techniques have demonstrated impressive performance in reducing inter-scanner variability in the image domain (Dewey et al., 2019;Guan et al., 2021;Hu et al., 2023;Modanwal et al., 2020). Nevertheless, few studies have explored the interpretability of harmonization models and the understanding of inter-scanner variability. Scarce availability of matched data across multiple sites also hinders comprehensive assessments of inter-scanner differences. The dearth of insight into the extent of inter-scanner variability highlights a need for interpretable image harmonization.

To address the above-mentioned challenges, this study aims to (1) characterize the effect of inter-scanner variability, (2) develop a procedure to harmonize cross-scanner images using the adapted statistical method to improve interpretability, (3) define a metric to qualitatively and quantitatively evaluate harmonization performance in an explainable manner, and (4) assess the impact of inter-scanner variability on image quality metrics and image-derived measures. To achieve these objectives, we proposed an analytic pipeline that incorporates ComBat in conjunction with a computer vision technique called superpixel parcellation (Achanta et al., 2012) to estimate the location and scale effect of inter-scanner variability at the image level on an individual basis. We adapted the paradigm of ComBat modeling to estimate the discrepancies of relative signal intensity distributions across scanners based on matched T1-weighted images. Through the proposed method, we decomposed the linear effect of inter-scanner variability in the image domain and further utilized ComBat-derived parameters to evaluate the harmonization performance for various well-established harmonization techniques. Moreover, we estimated various metrics to characterize multiple common image qualities and examined how image preprocessing procedures can benefit the reduction of inter-scanner variability. Additionally, the impact of inter-scanner variability on the T1-weighted image-derived measures was investigated in the study.

## 2 Materials & Methods

### 2.1 Study cohort & image acquisition

The study utilized a sample of 18 participants who were part of an ongoing project funded by the UH3 NS100608 grant awarded to Dr. J. Kramer and Dr. C. DeCarli (Wilcock et al., 2021). All participants (age: 68.0 [9.3] years; 10 females) were cognitively unimpaired and exhibited either a no-to-low (n = 9) or moderate-to-high (n = 9) degree of small vessel disease (SVD), as defined in a previous study (Lu et al., 2021). Each participant underwent neuroimaging examinations at four different 3T MRI scanners, including two Siemens systems (TIM Trio and Prisma), one Philips system (Achieva), and one General Electric (GE) system (750 W) according to the MarkVCID MRI protocol (Lu et al., 2021). The ethical approval followed the regulation in the MarkVCID consortium (Wilcock et al., 2021). For each participant, the matched image set was acquired within an interval of up to four months during which no biological changes in the brain were expected to occur. It is assumed that any observed differences between pairs of scans can be attributed solely to the scanner effects of the respective sites.

Specifically, for each subject, three-dimensional (3D) T1-weighted images were acquired on GE, Philips, Siemens-Prisma (SiemensP), and Siemens-Trio (SiemensT) scanners. For the GE scanner, the images were acquired by using the inversion-recovery prepared fast spoiled gradient recalled echo (IR-FSPGR) for Brain Volume Imaging (BRAVO) sequence with imaging parameters (1) field of view (FOV, mm): 256 × 256 × 172, (2) matrix size: 256 × 256 × 344, (3) flip angle: 10 degree, (4) repetition time (TR, ms): 9.5, (5) echo time (TE, ms): 3.7, (6) inversion time (TI, ms): 600, and (7) voxel size (mm): 1 × 1 × 0.5. For the Philips scanner, the images were acquired by using the multi-echo magnetization prepared rapid gradient echo (MPRAGE) sequence with imaging parameters (1) FOV: 256 × 256 × 176, (2) matrix size: 256 × 256 × 176, (3) flip angle: 7 degree, (4) TR: 2530, (5) TE: 1.66, (6) TI: 1300, and (7) voxel size: 1 × 1 × 1. For the SiemensP scanner, the images were also scanned by using the MPRAGE sequence with imaging parameters (1) FOV: 256 × 256 × 176, (2) matrix size: 256 × 256 × 176, (3) flip angle: 7 degree, (4) TR: 2530, (5) TE: 1.64, (6) TI: 1100, and (7) voxel size: 1 × 1 × 1. For the SiemensT scanner, the images were scanned by using the same imaging parameters and sequence as those implemented at the SiemensP scanner except TI = 1200. Notably, the TR shown for IR-FSPGR indicates the repetition time for each phase encoding step, whereas that shown for MPRAGE refers to the repetition time with respect to the nonselective inversion recovery pulse.

### 2.2 Framework for inter-scanner variability estimation and joint harmonization strategy

To characterize the location and scale effects of inter-scanner variability in T1-weighted images at the image level, we devised an analytic pipeline by incorporating the ComBat modeling and superpixel algorithm (Achanta et al., 2012). This pipeline, illustrated inFigure 1, encompasses four key steps: image preprocessing, spatial registration, superpixel parcellation, and ComBat modeling. Briefly, the pipeline takes cross-scanner matched data as input and applies preprocessing and spatial registration to standardize images in terms of dimensions, signal ranges, orientations, artifacts, etc. Individual averaged images then undergo superpixel parcellation to segment voxels into clusters. Scanner-related effects within the superpixels are estimated using ComBat modeling. For harmonization, the preprocessing procedure is combined with site effect removal using the group-level averaged ComBat parameters to eliminate inter-scanner variability. To evaluate harmonization performance, the procedure can be reapplied to the harmonized images, estimating any residual inter-scanner differences through the ComBat parameters.

**Fig. 1.**
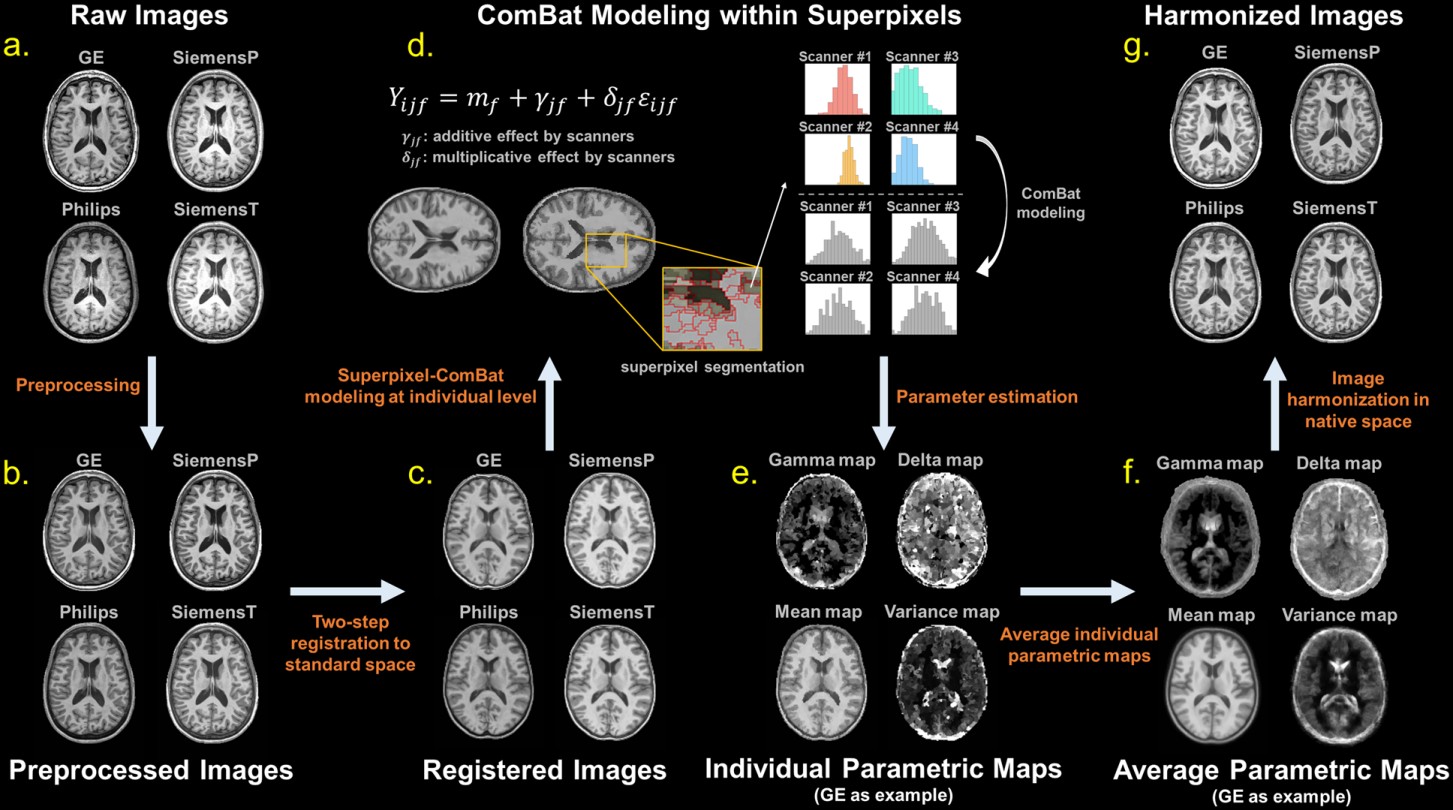
Analytic pipeline for estimating and harmonizing inter-scanner variability. (a) Matched T1-weighted images from four scanners were acquired. (b) Preprocessing using CAT12 was applied to the images to ensure basic image qualities. (c) The images were registered into a standard space through within- and between-subject spatial registration. (d) 3D superpixel segmentation was applied to individual averaged images, and the ComBat modeling was used to estimate the scanner effects within superpixels. (e) The estimated additive ($\gamma_{jf}$) and multiplicative ($\delta_{jf}$) effects characterize the location and scale effects of the inter-scanner variability on an individual basis. (f) Average parametric maps at the group level were estimated using cross-validation. (g) These average parametric maps were further used to perform site effect removal for harmonization.

#### 2.2.1 Image preprocessing

In practice, all T1-weighted images first went through a series of standard preprocessing (Fig. 1b) mainly including image resize, bias field correction, denoise, and global intensity normalization by using SPM12 (Ashburner et al., 2014) and CAT12 (Gaser et al., 2022) packages. The preprocessing steps were utilized to standardize basic image quality and fulfill the assumptions for ComBat model. To ensure compatibility of image dimension among the acquired images, a resizing procedure using B-spline interpolation was applied to the GE images to convert matrix size from 256 × 256 × 344 to 256 × 256 × 176 so that all analyzed images possessed the same voxel size (isotropic 1 mm^3^). To fulfill the unimodal assumption in ComBat estimation, the images underwent denoising using a spatial-adaptive non-local means filter (Manjón et al., 2010), which effectively eliminated noise while preserving the image edges. Next, the SPM-based bias field correction (Ashburner & Friston, 2005) was employed to eliminate signal inhomogeneity in T1-weighted imaging. To eliminate redundant clusters during superpixel parcellation, a background removal step was conducted, setting all voxels outside the head region to zero. Subsequently, global signal intensity normalization was employed (Alfaro-Almagro et al., 2018), rescaling the signal intensity across the entire image to a range from zero to one thousand, based on the minimum value and the 99^th^percentile of intensity values. By doing so, we were able to characterize the contrast difference between scanners. To estimate inter-scanner variability based on cross-scanner data, all images were aligned together in a standard space through a two-step spatial registration (Fig. 1c). Specifically, all within-subject T1-weighted images were registered together by using an affine transformation, and then the deformation-based registration was used to project all T1-weighted images onto the ICBM152 template in the MNI space (1.5 mm isotropic resolution) by using diffeomorphic registration algorithm with geodesic shooting (Ashburner & Friston, 2011). The hyperparameters of spatial registration (affine and SHOOT registration) were set according to the default settings used in the CAT12 (Gaser et al., 2022).

#### 2.2.2 Superpixel-ComBat modeling

The first-level inter-scanner variability was estimated on an individual basis. First, assuming that the average of cross-scanner images from a given subject can represent for that subject’s neuroanatomical structures, each subject’s cross-scanner images were averaged into a reference image that was used to performed superpixel parcellation. The superpixel technique segments an image into multiple clusters (called superpixels) based on similarity measures defined using perceptual features such as intensity and spatial distances (Achanta et al., 2012). The process involves generating superpixel centers and iteratively assigning pixels/voxels to their closest center while continuously updating the center locations. In this case, a 3D superpixel parcellation implemented by the simple linear iterative clustering algorithm (Achanta et al., 2012) was applied to the averaged image to generate image parcellation (Fig. 1d). Since we subsequently use voxels instead of subject outcome measures as observations in ComBat modeling, to ensure reliable estimation in cross-scanner images, a constraint was imposed during the superpixel segmentation process; each 3D superpixel was required to contain a minimum of 27 voxels, guaranteeing an adequate number of samples (Supplementary Material S1). As a result, each subject’s average image would be segmented into approximately 13,000 superpixels.

Next, we applied the ComBat model (Johnson et al., 2007;Torbati et al., 2021) to estimate the scanner effect within each 3D superpixel on an individual basis (Fig. 1d). ComBat models the data distribution as a linear combination of biological covariates and scanner effects, and applies adjustments to achieve harmonization across different sites or scanners (Torbati et al., 2021). By using superpixel parcellation, we can reduce the number of estimated parameters and meet the assumption of unimodal distribution for ComBat modeling compared to implementing voxel-wise ComBat (VoxelComBat) approach (Supplementary Material S2). In practice, the coordinates of segmented 3D superpixels were further applied to each of the cross-scanner images to sample voxels at the individual level. Within each superpixel of interest, the voxels were sampled as observations for ComBat modeling. The model would estimate the additive and multiplicative effects in terms of signal intensity within each superpixel. Because of using matched data, the modeling process would not need to consider biological covariates as in the ComBat implementation (Torbati et al., 2021). All parameters and scanner effects were estimated separately per subject. Thus, our modified model equation is:

$$Y_{ijf}=m_{f}+\gamma_{jf}+\delta_{jf}\varepsilon_{ijf},$$

where the value ($Y_{ijf}$) for each superpixel*f*, voxel*i*, and scanner*j*was modeled by the overall mean of superpixel ($m_{f}$), the additive location effect ($\gamma_{jf}$), and the multiplicative scale effect ($\delta_{jf}$), and the error term follows$\varepsilon_{ijf}\sim N(0,\;\sigma_{f}^{2})$. In practice, the$Y_{ijf}$was standardized to$Z_{ijf}$by

$$Z_{ijf}=\frac{Y_{ijf}-{\hat{m}}_{f}}{{\hat{\sigma }}_{f}},\;\;\text{Where}\;{\text{Z}}_{ijf}∼N(\gamma_{jf},\delta_{jf}^{2}).$$

The$\gamma_{jf}$and$\delta_{jf}$are assumed to follow$\gamma_{jf}\sim N(\mu_{j},\;\;\tau_{j}^{2})$and$\delta_{jf}^{2}\sim \text{Inverse Gamma}(\lambda_{j},\theta_{j})$. To remove the scanner effect, the harmonization can be achieved by calculating

$$Y_{ijf}^{*}=\frac{{\hat{\sigma }}_{f}}{{\hat{\delta }}_{jf}}\left(Z_{ijf}-{\hat{\gamma }}_{jf}\right)+{\hat{m}}_{f},$$

where the “hat” symbol indicates estimated quantities.

With this model, we estimated additive and multiplicative terms representing inter-scanner variability for each superpixel, generating individual parametric maps for the scanner effect (Fig. 1e). The parametric maps were then averaged across subjects to produce general scanner effect maps characterizing the inter-scanner differences (Fig. 1f). The overall procedure was implemented using MATLAB R2022a (The MathWorks Inc., Natick, MA, USA). Besides representing the scanner effect, the parametric maps can be the basis of the image harmonization procedure (Fig. 1g). Since the estimated average parametric maps were initially in the standard space, we transformed them to each subject’s native space using that subjects’ inverse deformation field. Scanner effect was removed from cross-scanner images by subtracting the additive maps and then dividing by multiplicative maps (i.e., linear image harmonization). We can achieve retrospective image harmonization by combining image preprocessing steps and the linear site effect removal as a joint harmonization strategy, namely SP-ComBat approach. To avoid biasing the harmonization evaluation due to circular analysis, we performed the step of averaging parametric maps using a leave-one-out strategy (Wong, 2015). Specifically, the ComBat parameters used to harmonize the images of a certain subject were estimated using a held-out sample set that excluded that particular subject (Fig. 1f). We also implemented VoxelComBat (Supplementary Material S2) for comparison with our proposed SP-ComBat approach. The source code of the proposed SP-ComBat implemented with MATLAB can be found in our GitHub repository (https://github.com/ChangleChen/SPComBat).

### 2.3 Image quality assessments

We investigated characteristics of general image quality metrics for T1-weighted images over scanners using an open-access analytic toolbox called MRIQC package (Esteban et al., 2017). Image quality metrics mainly assess voxel size, signal-to-noise ratio (SNR), contrast-to-noise ratio (CNR), signal uniformity, and artifact presence (Esteban et al., 2017;Rosen et al., 2018). Specifically, the SNR metrics were measured for each primary tissue type (i.e., GM, WM, and CSF); the calculation was defined as the mean signal intensity in a certain tissue type divided by the within-tissue variance. Higher values indicate better signal quality. The CNR was estimated to assess the distinction between the tissue distributions of GM and WM. Higher values indicate better GM-WM contrast. The coefficient of joint variation (CJV) of GM and WM, suggested as an objective function for optimizing intensity non-uniformity correction algorithms (Ganzetti et al., 2016), was calculated to assess the extent of head motion and signal inhomogeneity artifacts. Higher values are associated with the existence of substantial head motion and significant inhomogeneity artifacts. The white matter to maximum intensity ratio (WM2MAX) was assessed by measuring the median intensity of the WM mask relative to the 95^th^percentile of the overall WM intensity distribution. This captures the presence of outlier values caused by hyper-intensity of carotid vessels and fat, representing biological signal inhomogeneity within WM. The values should fall within a specific range approximately between 0.6 and 0.8 (Esteban et al., 2017). The image quality assessments were performed before and after the SP-ComBat procedure.

### 2.4 Evaluation of image harmonization

#### 2.4.1 Image harmonization techniques

We leveraged harmonized images from our previous work (Torbati et al., 2023) to compare different harmonization strategies (described inSection 2.4.2). Specifically, the same T1-weighted images were harmonized by two established deep learning-based image harmonization techniques, namely CALAMITI (Dewey et al., 2020) and MISPEL (Torbati et al., 2023), as well as the methods proposed in this study (i.e., SP-ComBat and VoxelComBat). CALAMITI utilizes an autoencoder architecture that integrates encoders for content and style, a generator, and a batch discriminator to perform image-to-image translation between domains (Dewey et al., 2020). MISPEL leverages a U-net architecture that harmonizes images across multiple batches by performing content-style disentanglement across domains (Torbati et al., 2023). These four image harmonization techniques were further evaluated by retrospective metrics listed in the next section. Notably, we performed SP-ComBat harmonization by removing average additive and multiplicative terms from cross-scanner images using leave-one-out validation for fair comparison (Methods 2.2). However, for VoxelComBat, since the parametric maps were directly estimated at the group level, potential biases may exist.

#### 2.4.2 Retrospective evaluation metrics at the image level

The proposed analytical procedure can also reflect the residual of inter-scanner variability after harmonization by re-estimating ComBat-derived parametric maps, serving as a metric to evaluate the effectiveness of image harmonization. The additive effect captures the relative mean difference of voxel intensities between scanners, and the multiplicative effect represents the variability of intensities. A well-harmonized image should demonstrate additive terms close to zero and multiplicative terms close to one, indicating minimal deviation from the joint distribution (i.e., approaching to a unified standard normal distribution). Besides, the image harmonization techniques were assessed using structural similarity index (SSIM) (Wang et al., 2004), a common index for assessing image similarity between cross-scanner images given the luminance, contrast, and structural aspects, evaluating the presence of similar anatomy between matched images (Wang et al., 2004). Higher values of SSIM denote more structurally similar between images. Compared to SSIM, the SP-ComBat can jointly evaluate the distances of signal distributions relative to a joint distribution among scanners. This conceptual improvement is analogous to the extension from statistical*t*tests to analysis of variance (ANOVA). Furthermore, to evaluate the scanner effect residuals at the image level, we built convolutional neural network (CNN) classifiers to predict the scanner labels directly based on the harmonized images, which provided a robust assessment of harmonization methods. It is hypothesized that the trained CNN classifiers should accurately identify the scanner source from raw T1-weighted images while their predictive accuracy would significantly diminish when applied to well-harmonized images. Experiments were conducted under five conditions: raw (unharmonized), CALAMITI, MISPEL, VoxelComBat, and SP-ComBat images, with all images registered to the MNI space. In practice, the subjects were randomly divided into training (N = 9) and test (N = 9) sets, and 2D CNN classifiers were trained under each condition to predict the scanner source using 2D axial slices sampled from brain volumes. The details of this evaluation were provided inSupplementary Material S3.

In addition to assessing inter-scanner variability at the image level in terms of image characteristics, we performed voxel-based morphometry to investigate how the scanner effect would impact the biological estimates (e.g., the tissue volume of the brain) at the feature level. The CAT12 toolbox was used to estimate volumetric measures of GM since it is the most common target tissue class of research interest (Gaser et al., 2022). We further divided the GM class into five main neuroanatomical regions, including the frontal, parietal, occipital, temporal, and limbic regions based on the LPBA40 atlas to observe regional differences related to inter-scanner variability (Shattuck et al., 2008). The detailed results for individual regions were also provided in theSupplementary Materials.

### 2.5 Statistical analysis

To quantitatively analyze the location and scale effects of relative signal intensity over scanners, we applied tissue masks involving GM, WM, and CSF to individual parametric maps to sample tissue-specific additive and multiplicative effects. These masks were generated by the common study-specific template and used for all tissue-wise comparison for consistent assessments. The signal intensity distributions of well-harmonized images should have a zero additive effect and a multiplicative effect of one. Thus, one-sample*t*tests were used to test if the additive and multiplicative effects were significantly different from those normative values over scanners. Additionally, the SSIM was tested between raw and harmonized images with paired*t*tests while the classification accuracy of scanner prediction was tested between raw and harmonized images with two-sample*t*tests due to the independence between CNN classifiers. Regarding image quality metrics, paired*t*tests were conducted to evaluate the statistical difference between scanners.

To evaluate the impact of inter-scanner variability on neuroanatomical measures (i.e., volumetric estimates), we estimated the coefficients of variation (CV) for neuroanatomical measures across scanners in major GM regions. Higher CV indicates greater uncertainty in the estimation of GM volume across scanners. We assessed how main image preprocessing steps, that is, denoising and debiasing (bias field correction), and linear image harmonization would affect the CV for GM volume measures. We calculated the CVs in the following conditions: (1) raw images (without denoising and debiasing procedures before running voxel-based morphometry), (2) denoised images (without debiasing), (3) debiased images (without denoising), (4) denoised plus debiased images, and (5) harmonized images (processed by complete SP-ComBat procedure). We compared the CV estimates derived from the raw images with those from other conditions using paired*t*tests, assessing whether these main image preprocessing steps could reduce the inter-scanner variability in terms of GM volumetric measures. We also compared the raw and SP-ComBat harmonized images to those harmonized by CALAMITI, MISPEL, and VoxelComBat.

The participants in this study consisted of individuals with low and high risk of SVD. Potential atrophies of the hippocampus, parahippocampal gyrus, and inferior temporal gyrus have been reported in SVD (Jokinen et al., 2012), so we targeted these regions according to the LPBA40 atlas (Shattuck et al., 2008) and estimated the effect size of SVD using Cohen’s d, which represents the biological effect of SVD for those affected GM regions. Differences in effect size of SVD were evaluated among harmonization approaches.

## 3 Results

### 3.1 Location and scale effects of inter-scanner variability

**Fig. 2.**
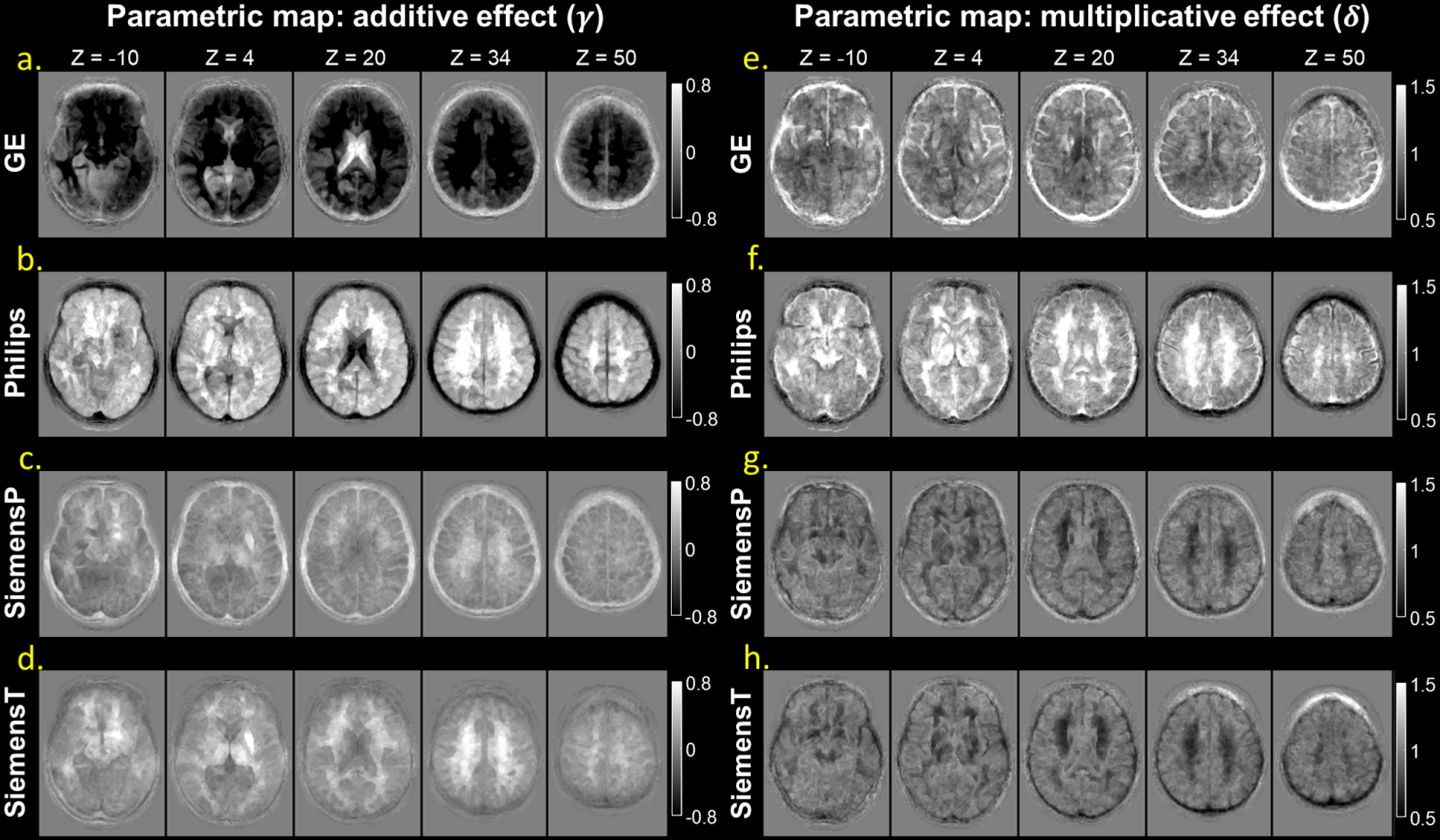
Estimated maps demonstrating inter-scanner variability in the MNI space. The maps depict the additive (γ) and multiplicative (δ) effects specific to each scanner; GE (a & e), Philips (b & f), SiemensP (c & g), and SiemensT (d & h). The former represents the shift in signal distributions relative to the average (i.e., location effect), while the latter demonstrates the spread of the distribution (i.e., scale effect). Positive or negative deviations in the additive effect denote signal intensities greater or less than the average, respectively. Values above or below one for the multiplicative effect signify variation in signal intensities that is expanded or contracted compared to the average (a standard normal distribution), respectively. The slices are sequentially displayed from inferior (z = -10) to superior (z = 50) level in the brain.

Figure 2visualized the estimated additive (γ) and multiplicative (δ) effects of inter-scanner signal distributions. The additive maps revealed more pronounced signal deviations at the GE and Philips scanners (Fig. 2a & 2b) compared to SiemensP and SiemensT (Fig. 2c & 2d). Notably, differences in signal intensity were more evident in the WM and CSF. In the multiplicative maps, Philips exhibited greater signal variation, particularly in WM (Fig. 2f). Additionally, signal heterogeneity near the skull and pial regions may suggest the presence of a residual bias field (Fig. 2e & 2hat z = 50).

In the quantitative analysis of tissue-specific location and scale effects, we found that, at the GE scanner, the additive effects in the GM and WM were significantly lower than the normative value while the CSF showed significantly positive deviation (Supplementary Material S4). On the contrary, the additive effect in the CSF at the Philips scanner showed a significantly negative deviation. The additive effects at the SiemensP scanner were not significant, suggesting that the locations of signal distribution at SiemensP were close to the joint distribution of the average. However, the additive effects in the GM and WM at the SiemensT scanner were significantly greater than zero. Most of tissue-specific multiplicative effects significantly deviated from the normative value; the multiplicative effects observed at the Philips scanner were significantly greater than those of other scanners, whereas the values observed at SiemensP and SiemensT scanners were significantly less than the average.

### 3.2 Image quality metrics across scanners

The image quality metrics of the cross-scanner images before image processing (i.e., raw image) reported inTable 1reveal notable variations in the SNR among the major tissue types. Specifically, the SNR in GM was relatively higher at GE scanner compared to the rest of the scanners. However, Siemens scanners demonstrated higher SNR in WM compared to Philips and GE. The CNR between GM and WM generally exhibited comparable values across the scanners; nevertheless, there were statistically significant differences with respect to CNR at GE and SiemensP. The CJV values reflecting field inhomogeneity are also reported inTable 1. We found noticeable variations in CJV across different scanners.

**Table 1.** Image quality metrics of cross-scanner T1-weighted images and their comparisons.

	Metrics	GE	Philips	SiemensP	SiemensT	GE vs. Philips	GE vs. SiemensP	GE vs. SiemensT	Philips vs. SiemensP	Philips vs. SiemensT	SiemensP vs. SiemensT
Raw images	SNR-GM	13.50 (1.02)	11.04 (0.90)	11.23 (0.99)	12.77 (1.28)	**2.46 (0.84)**	**2.27 (0.71)**	**0.73 (0.70)**	-0.19 (0.91)	**-1.74 (1.01)**	**-1.54 (0.65)**
	SNR-WM	17.74 (2.50)	18.26 (2.18)	23.54 (3.17)	23.09 (3.78)	-0.52 (1.85)	**-5.80 (2.01)**	**-5.35 (2.93)**	**-5.28 (2.35)**	**-4.83 (2.72)**	0.45 (1.93)
	SNR-CSF	2.04 (0.32)	2.25 (0.49)	2.54 (0.50)	3.34 (0.70)	-0.22 (0.50)	**-0.50 (0.35)**	**-1.30 (0.52)**	**-0.29 (0.34)**	**-1.09 (0.46)**	**-0.80 (0.23)**
	CNR-GM/WM	3.43 (0.24)	2.99 (0.19)	3.41 (0.23)	3.14 (0.24)	**0.44 (0.17)**	0.02 (0.21)	**0.29 (0.18)**	**-0.41 (0.19)**	**-0.15 (0.15)**	**0.27 (0.15)**
	WM2MAX	0.51 (0.07)	0.75 (0.06)	0.68 (0.07)	0.72 (0.09)	**-0.23 (0.06)**	**-0.16 (0.06)**	**-0.20 (0.07)**	**0.07 (0.05)**	**0.03 (0.05)**	**-0.04 (0.04)**
	CJV	0.39 (0.03)	0.45 (0.03)	0.39 (0.03)	0.42 (0.03)	**-0.06 (0.02)**	-0.01 (0.02)	**-0.03 (0.03)**	**0.05 (0.02)**	**0.02 (0.02)**	**-0.03 (0.02)**
Harmonized images	SNR-GM	13.04 (0.89)	13.36 (0.75)	11.76 (0.79)	12.43 (1.05)	-0.32 (0.68)	**1.28 (0.75)**	**0.60 (0.91)**	**1.59 (0.74)**	**0.92 (0.77)**	**-0.67 (0.65)**
	SNR-WM	30.40 (3.65)	28.21 (3.72)	31.34 (3.90)	30.97 (5.13)	**2.19 (2.29)**	-0.94 (1.79)	-0.58 (2.34)	**-3.13 (2.99)**	**-2.76 (2.95)**	0.36 (2.54)
	SNR-CSF	3.41 (0.45)	4.38 (0.83)	3.42 (0.74)	3.84 (0.90)	**-0.97 (0.71)**	-0.01 (0.44)	**-0.43 (0.62)**	**0.96 (0.62)**	**0.54 (0.60)**	**-0.42 (0.28)**
	CNR-GM/WM	5.04 (0.26)	4.66 (0.28)	4.74 (0.21)	4.77 (0.29)	**0.38 (0.19)**	**0.30 (0.24)**	**0.27 (0.20)**	-0.08 (0.28)	-0.11 (0.27)	-0.03 (0.23)
	WM2MAX	0.84 (0.06)	0.90 (0.04)	0.84 (0.06)	0.86 (0.06)	**-0.06 (0.04)**	-0.00 (0.03)	-0.02 (0.04)	**0.06 (0.03)**	**0.04 (0.02)**	**-0.02 (0.02)**
	CJV	0.27 (0.01)	0.30 (0.02)	0.28 (0.01)	0.28 (0.02)	**-0.03 (0.01)**	-0.01 (0.02)	-0.01 (0.01)	**0.02 (0.02)**	**0.02 (0.02)**	-0.00 (0.02)

Note: (1) The values in each cell indicate the mean and standard deviation of image quality metrics. (2) Harmonized images refer to those processed with SP-ComBat procedure. (3) Bold text indicates the comparison with significance*p*< 0.05. (4) The definition of noise in SNR is the within-tissue variance. (5) CNR is defined between GM and WM.

Abbreviation: SNR: signal to noise ratio, CNR: contrast to noise ratio, WM2MAX: white matter to maximum intensity ratio, CJV: coefficient of joint variation, GM: gray matter, WM: white matter, CSF: cerebrospinal fluid.

Image quality metrics of harmonized images by SP-ComBat procedure are shown in bottom section ofTable 1. The SNR in WM and CSF was substantially improved after the harmonization. Although the SNR in GM was not significantly improved, it became more consistent among scanners. Furthermore, the CNR between GM and WM demonstrated significant, consistent improvement for all scanners after the harmonization process. The WM2MAX index also showed increased consistency across scanners, likely attributable to the enhanced SNR in WM. Moreover, the CJV metric exhibited significant reduction for all scanners, suggesting successful elimination of signal inhomogeneity. These image quality metrics showed substantial improvement and greater consistency across scanners following image preprocessing and scanner effect removal by SP-ComBat. Nevertheless, certain metrics still exhibited significant scanner differences after harmonization (Table 1, bold text in the lower right section).

### 3.3 Evaluation of image harmonization

#### 3.3.1 Proposed harmonization strategy

The qualitative results of SP-ComBat harmonization approach are shown inFigure 3. In comparison to the original raw images, the harmonized images exhibited more consistent signal profiles and similar contrasts across scanners. As depicted inFigure 4, the re-estimated additive and multiplicative effects were closer to zero and one, respectively, in comparison to the original parameters (Fig. 2), indicating a substantial reduction of the scanner effect. However, we still observed residual signal deviation in WM at Philips, SiemensP, and SiemensT (Fig. 4b-d). In the quantitative analysis of tissue-specific scanner effects, it was observed that the re-estimated additive effects of GM and WM at GE and SiemensT scanners became statistically insignificant, while the CSF still demonstrated significant deviation (Supplementary Material S5). Regarding the multiplicative effects, the signal spread in GM and WM was significantly reduced after harmonization; however, the signal distributions in CSF were not thoroughly corrected for all scanners (Supplementary Material S5).

**Fig. 3.**
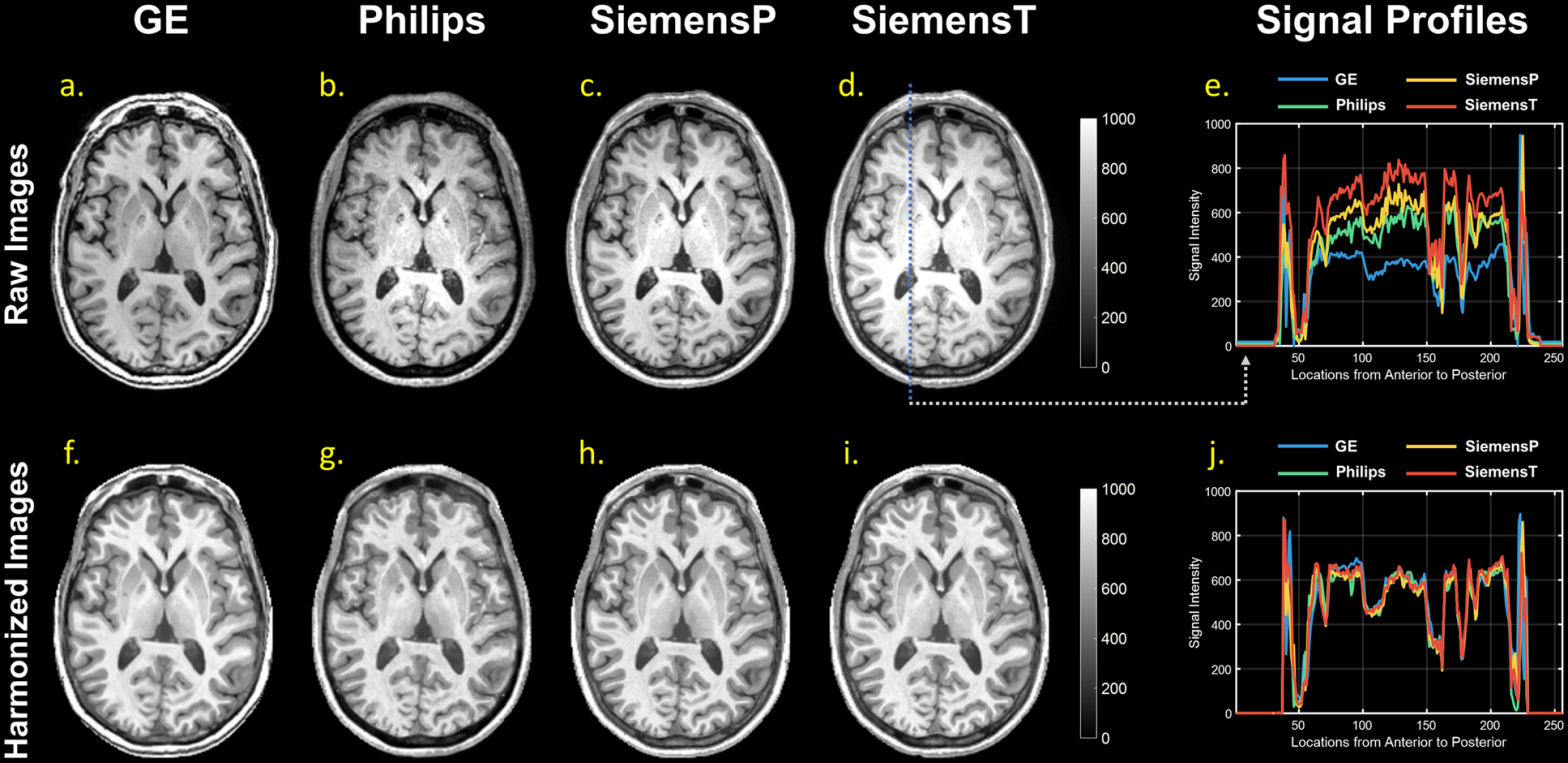
Visualization of raw images (a-d) and harmonized images (f-i) across scanners. Harmonization here refers to the SP-ComBat procedure. The signal profiles sampled along with a certain anterior-posterior axis are provided for visual comparison (e & j). To provide fair visual comparison, all images underwent the global signal intensity normalization to transform voxel values in to the range between 0 and 1000.

**Fig. 4.**
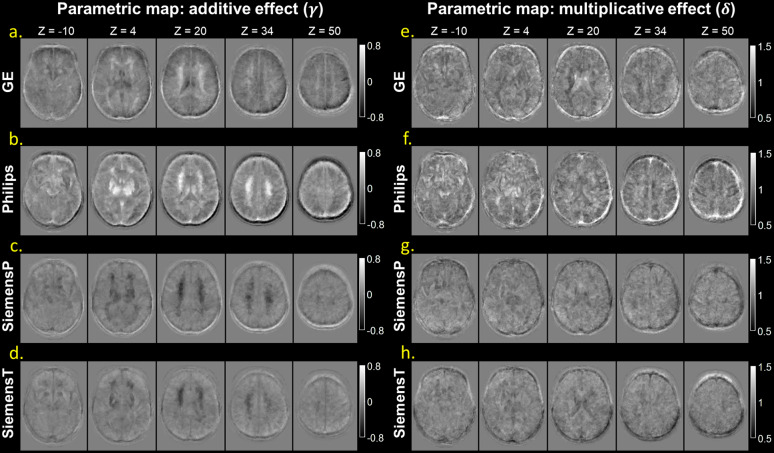
Estimated maps after SP-ComBat harmonization. The maps demonstrate the additive effect (γ) and multiplicative effect (δ) specific to each scanner after harmonization with leave-one-out cross validation (additive: a-d, multiplicative: e-h). Compared to the parametric maps shown inFigure 2, the signal deviation and variation were eliminated after linearly removing the scanner effect. The slices are sequentially displayed from inferior (z = -10) to superior (z = 50) level in the brain.

#### 3.3.2 Evaluation metrics for image harmonization

**Fig. 5.**
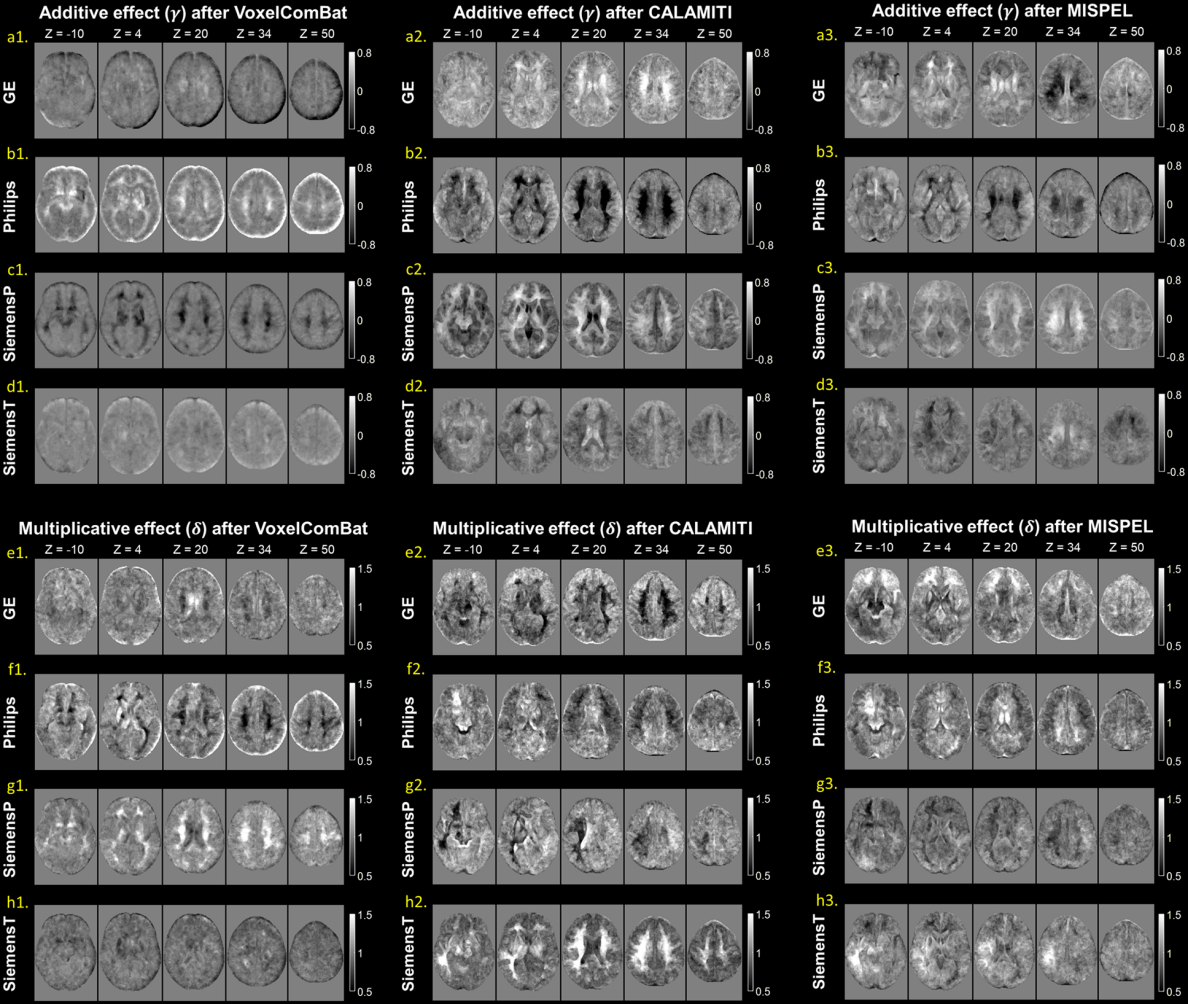
Estimated maps of additive and multiplicative effects after harmonization. The additive effect (a1-d3) captures the relative signal deviation from the joint distribution, and the multiplicative effect (e1-h3) represents the variability of intensities. The slices are sequentially displayed from inferior (z = -10) to superior (z = 50) level in the brain.

**Fig. 6.**
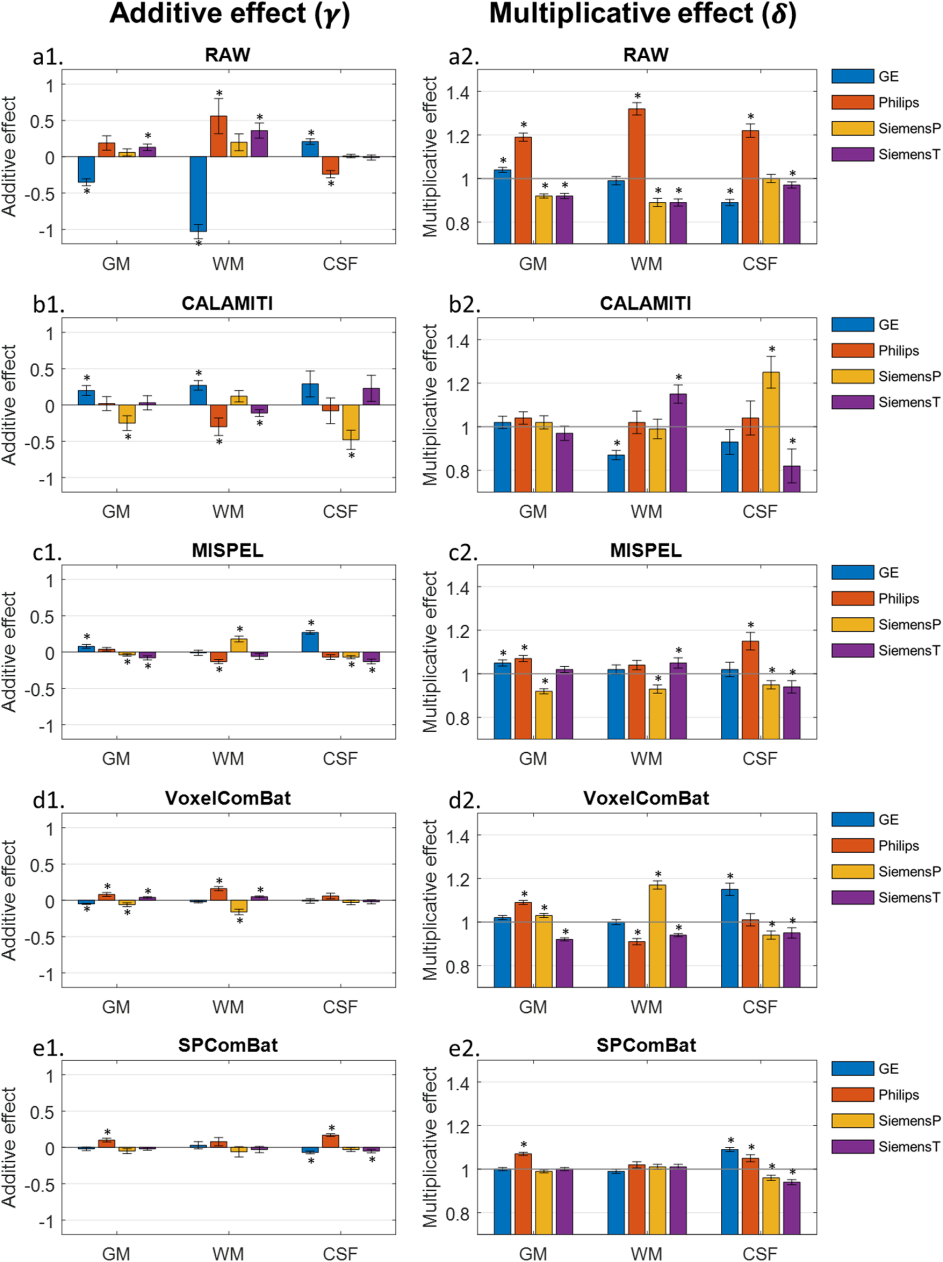
Tissue-specific additive (a1-e1) and multiplicative (a2-e2) effects before and after harmonization. The effect values derived from different harmonization methods were sampled by using common tissue-specific masks across scanners and subjects. Notably, the raw condition here (i.e., sampled fromFig. 2) refers to the images only with basic image preprocessing mentioned inSection 2.2. Error bars indicate the standard error of mean. Asterisk used to indicate the comparison relative to the hypothetical values with significance*p*-values < 0.05. Abbreviation: GM: gray matter, WM: white matter, CSF: cerebrospinal fluid.

The parametric maps derived from CALAMITI, MISPEL, and VoxelComBat are also visualized inFigure 5, and the tissue-specific parameters of harmonized images are illustrated inFigure 6. Regarding signal deviations quantified by additive effects, for deep learning approaches, CALAMITI tended to over-correct the contrast difference (e.g., GE and Philips) compared to the raw images (Fig. 5a2-d2&Fig. 6b1). While some residual contrast differences remained, MISPEL generated more balanced tissue contrasts compared to CALAMITI (Fig. 5a3-d3&Fig. 6c1). VoxelComBat seems to provide comparable results to SP-ComBat, but there were still noticeable residuals of inter-scanner differences remaining around tissue boundaries (Fig. 5a1-d1&Fig. 6d1). SP-ComBat method with the leave-one-out strategy generated least residual contrast differences compared to others (Fig. 4&Fig. 6e1); however, since it utilized parametric maps to harmonize images, the performance assessed by the estimated parameters could be optimistic (as with VoxelComBat).

**Fig. 7.**
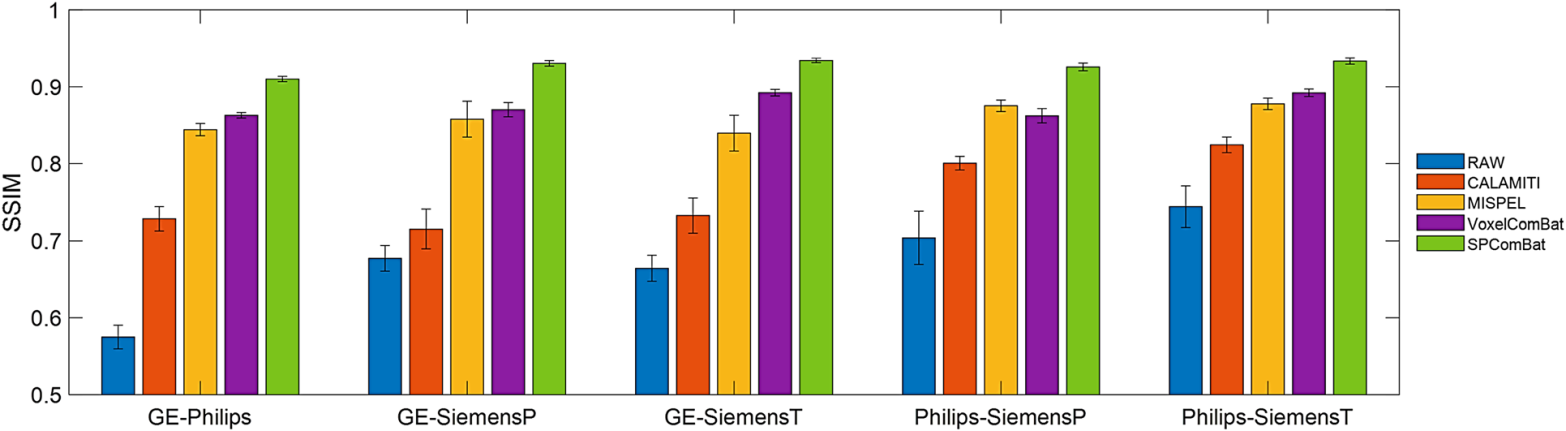
Structural similarity index (SSIM) after harmonization. The SSIM was calculated between pairs of images (e.g., GE-Philips). Compared to the raw, all harmonized images show the significant difference (*p*-values < 0.05) except CALAMITI for GE-SiemensP and SiemensP-SiemensT. Error bars indicate the standard error of mean.

The retrospective evaluation metric, SSIM, was also applied to the harmonized images (Fig. 7). After harmonization, the image similarities between cross-scanner images were significantly improved by all methods (*p*< 0.05) except CALAMITI for GE-SiemensP and SiemensP-SiemensT. The result was in line with the observations from parametric maps. Nevertheless, compared to using an overall metric to evaluate image similarity as retrospective evaluation for image harmonization, we considered that using ComBat-derived parameters is more interpretable and able to jointly evaluate cross-scanner images.

**Fig. 8.**
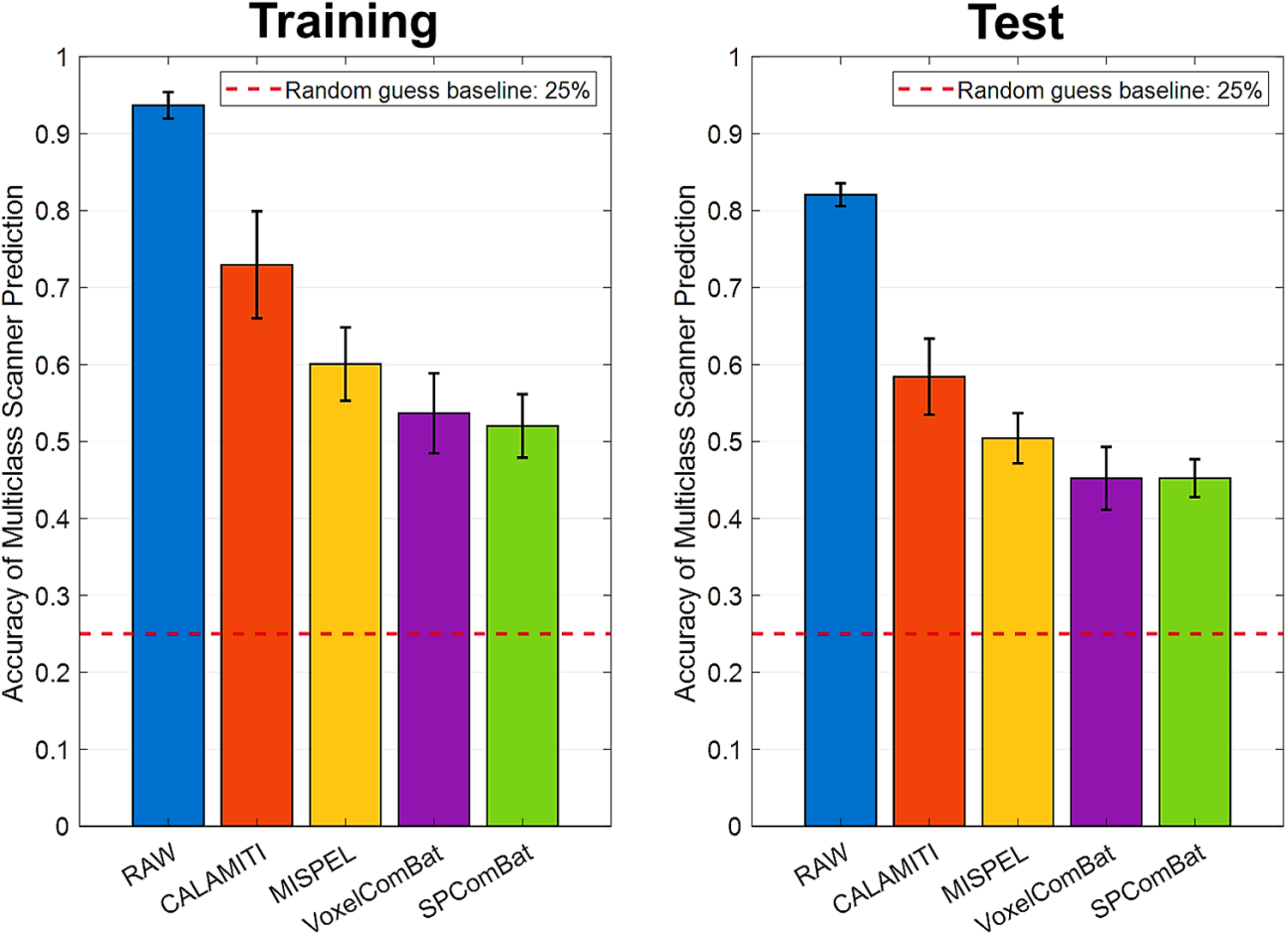
Multiclass classification accuracy for scanner prediction after harmonization. The accuracy was calculated on training and test sets for each condition. Ideally, a well-harmonized method can significantly lower the prediction performance down close to the accuracy of 25%, indicating the random guess in four scanner sources. Compared to the raw, all harmonized images show the significant accuracy downgrade (*p*-values < 0.001).

We also leveraged CNN-based image classifiers to predict scanner effect residuals in the harmonized images. The results indicated that the image classifiers can accurately predict the scanner sources in the unharmonized condition, achieving a training accuracy of 0.937 and a test accuracy of 0.821 (Fig. 8). All harmonization methods significantly reduced classification accuracy in both the training and test sets (all*p*-values < 0.001) compared to the unharmonized one, demonstrating successful harmonization. However, compared to CALAMITI (training accuracy: 0.730, test accuracy: 0.584) and MISPEL (training accuracy: 0.601, test accuracy: 0.504), the accuracy metrics for VoxelComBat (training accuracy: 0.537, test accuracy: 0.452) and SP-ComBat (training accuracy: 0.520, test accuracy: 0.452) were closer to the random guess baseline of 25%, indicating a more ideal harmonization outcome.

### 3.4 Impact of inter-scanner variability on neuroanatomical measures

The impact of main image preprocessing steps and site effect removal achieved by SP-ComBat on the CV of GM volume across scanners was assessed by four processing levels inFigure 9a. Compared to the raw, preprocessing with denoising only did not significantly reduce the CV. However, preprocessing with bias correction resulted in averagely 21.9% CV reduction over regions compared to the raw; the reductions were significant (*p*< 0.05) in the parietal, occipital, and limbic regions (23.7%, 33.3%, and 26.6%, respectively). The combination of both preprocessing steps achieved the comparable level of CV reduction compared to that solely using bias correction. Furthermore, combining preprocessing steps as well as site effect removal (i.e., SP-ComBat procedure) can significantly, in average, reduce the CV by 40.3% compared to the raw (*p*< 0.001).

**Fig. 9.**
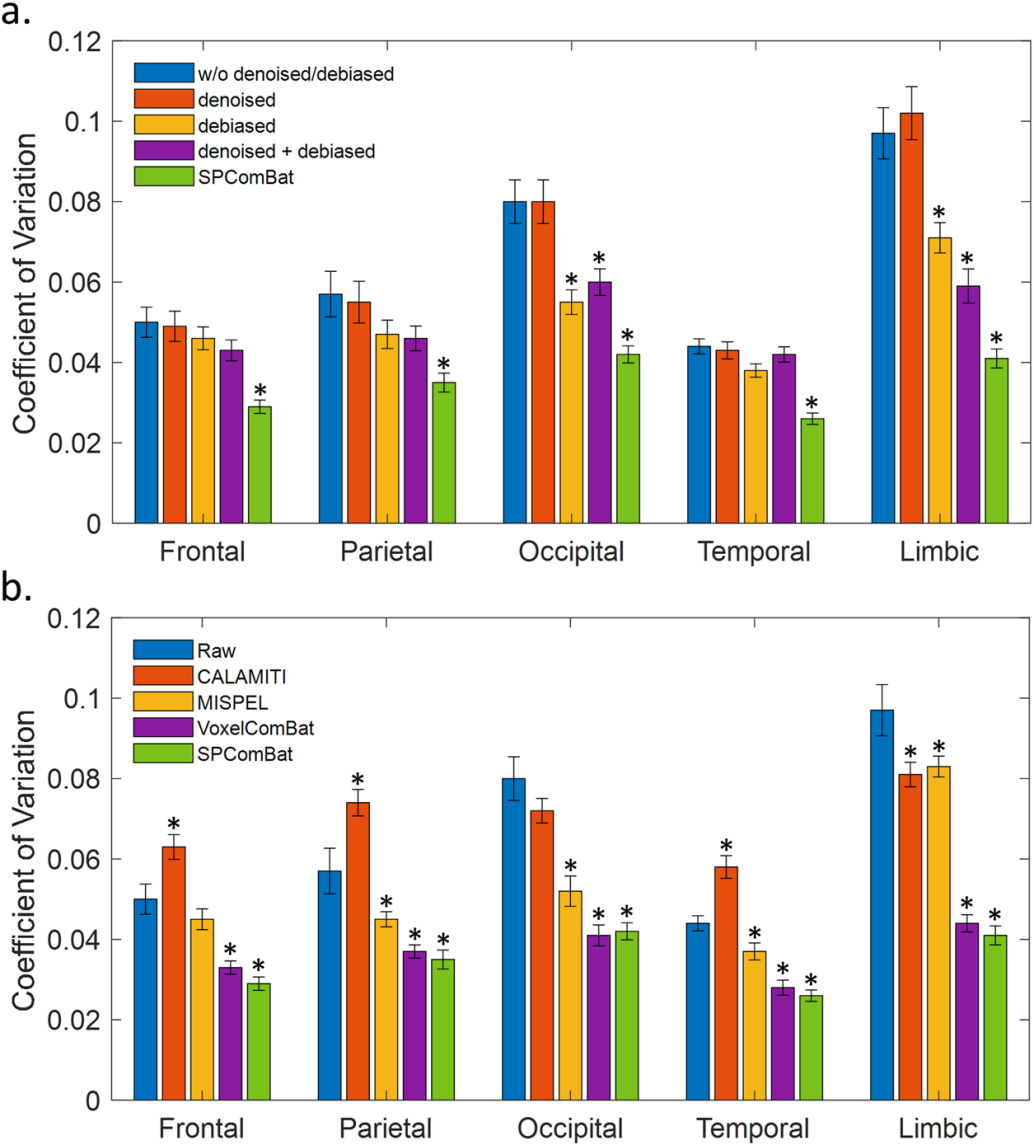
Coefficients of variation for major regional gray matter volume measures under different preprocessing conditions (a) and harmonization methods (b). Error bars indicate the standard error of mean. Asterisk used for indicating significance between raw image and other conditions: *:*p*-values < 0.05. Definition in preprocessing conditions: (1) raw images (without denoising/debiasing before running voxel-based morphometry), (2) denoised images (without debiasing), (3) debiased images (without denoising), and (4) images processed with both denoise and debias.

On the other hand, regarding different harmonization methods, most of them can significantly reduce the CV in major GM regions (*p*< 0.05) except CALAMITI (Fig. 9b). This may suggest that, although deep learning-based harmonization approaches can significantly improve overall spatial consistency of cross-scanner images (Fig. 7), the uncertainty of region-specific estimates may still exist. However, by combining image preprocessing based on domain knowledge with adapted statistical harmonization, we can significantly improve the consistency of GM estimates. The detailed CVs of individual GM regions were reported in theSupplementary Material S6.

### 3.5 Impact of inter-scanner variability on biological signals

**Fig. 10.**
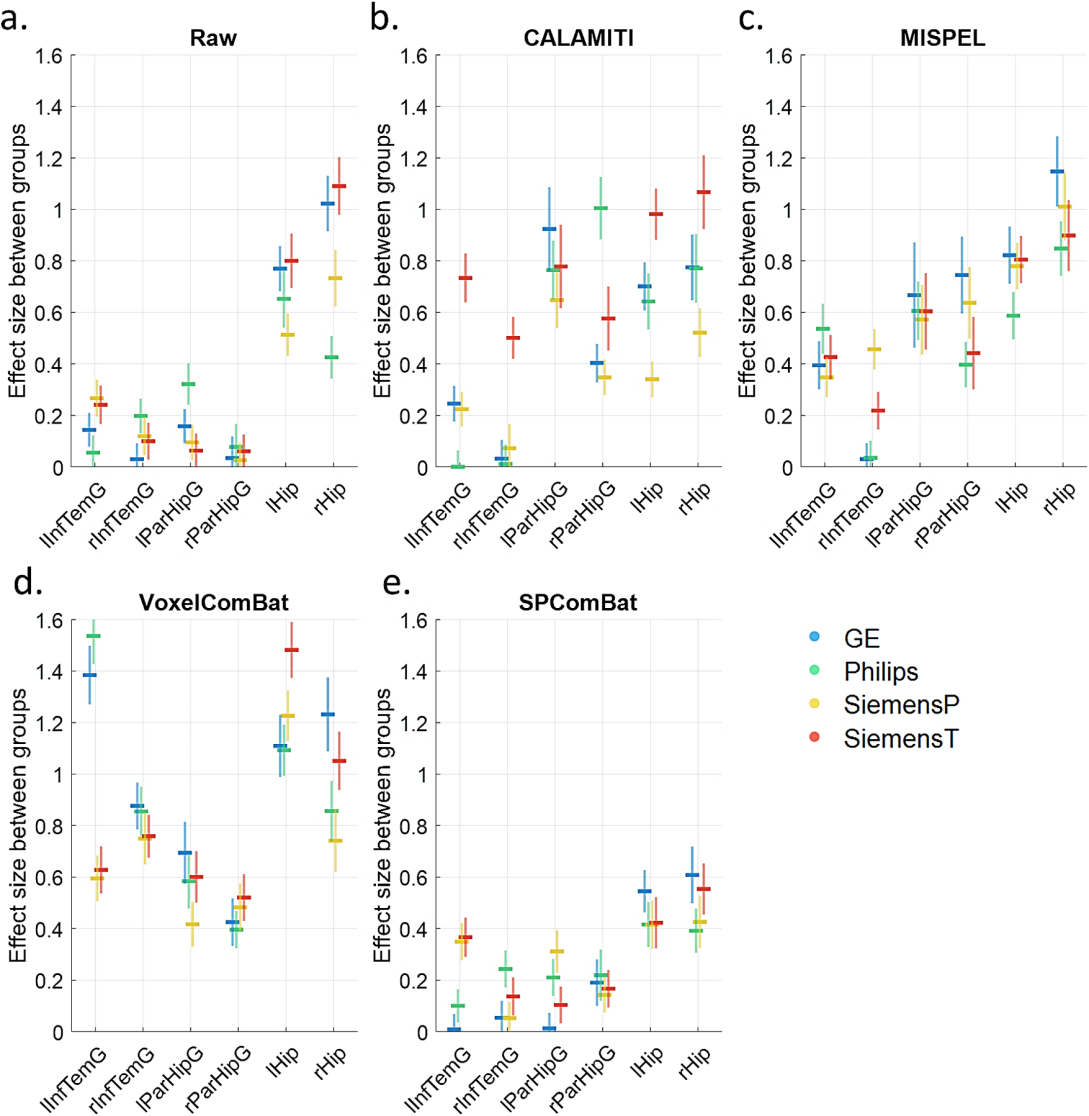
Effect size estimates of disease effect over scanners derived from raw images (a) and harmonized images (b-e). Horizontal lines represent the effect size estimates of disease, and vertical lines indicate their standard deviations obtained through bootstrapping. The blue, green, yellow, and red colors indicate GE, Philips, SiemensP, and SiemensT, respectively. Abbreviation: InfTemG: inferior temporal gyrus, ParHipG: parahippocampal gyrus, Hip: hippocampus. The prefixes “l” and “r” in the anatomical abbreviations indicate the left and right sides, respectively.

Cohen’s d values, representing the effect size between subjects with low and high risk of SVD, in six regions of interest (bilateral hippocampi, parahippocampal gyri, and inferior temporal gyri) are shown inFigure 10for raw and harmonized images. We observed that the Cohen’s d values in the raw condition (Fig. 10a) were scattered and relatively low among scanners, indicating discrepant and potentially under-estimated effect size of disease risks (mean [SD] of Cohen’s d: left inferior temporal gyrus (lInfTemG): 0.18 [0.10], right inferior temporal gyrus (rInfTemG): 0.11 [0.07], left parahippocampal gyrus (lParHipG): 0.16 [0.12], right parahippocampal gyrus (rParHipG): 0.05 [0.02], left hippocampus (lHip): 0.68 [0.13], right hippocampus (rHip): 0.82 [0.30]). After performing deep learning harmonization with CALAMITI and MISPEL, the effect size was increased across regions (Fig. 10b & 10c). However, with CALAMITI, the discrepancy of effect size among scanners seemed to increase (Cohen’s d: lInfTemG: 0.30 [0.31], rInfTemG: 0.15 [0.23], lParHipG: 0.78 [0.11], rParHipG: 0.58 [0.30], lHip: 0.67 [0.26], rHip: 0.78 [0.22]). MISPEL appeared to provide an enhanced effect size with less scanner differences in a more balanced manner (Cohen’s d: lInfTemG: 0.43 [0.08], rInfTemG: 0.19 [0.20], lParHipG: 0.61 [0.04], rParHipG: 0.56 [0.16], lHip: 0.75 [0.11], rHip: 0.98 [0.13]). Regarding VoxelComBat, the effect size appeared to increase as well, but it was scattered among scanners (Fig. 10d), indicating inconsistent estimates (Cohen’s d: lInfTemG: 1.04 [0.49], rInfTemG: 0.81 [0.07], lParHipG: 0.57 [0.12], rParHipG: 0.46 [0.06], lHip: 1.23 [0.18], rHip: 0.97 [0.22]). SP-ComBat seemed to reduce scanner differences the best compared to the other approaches, suggesting it can enhance the consistency of biological signals across scanners (Cohen’s d: lInfTemG: 0.21 [0.18], rInfTemG: 0.12 [0.09], lParHipG: 0.16 [0.13], rParHipG: 0.18 [0.03], lHip: 0.45 [0.06], rHip: 0.49 [0.10]). However, SP-ComBat was more conservative in reflecting the biological signals related to SVD, showing smaller effect size estimates across regions (Fig. 10e).

## 4 Discussion

In this study, we have developed an analytic pipeline to estimate the location and scale effects of inter-scanner variability at the image level and applied it to achieve image harmonization. This pipeline integrates a 3D superpixel parcellation algorithm and ComBat modeling (i.e., SP-ComBat) in which the model estimates an additive effect representing the shift in relative signal intensity and a multiplicative effect signifying the degree of signal variation. This adapted statistical harmonization strategy can successfully reduce the inter-scanner variability in an interpretable manner. We further demonstrated the application of using the additive and multiplicative terms to assess the inter-scanner variability residual after image harmonization. Additionally, we characterized various image quality and evaluation metrics of T1-weighted images affected by inter-scanner variability. In the*ad-hoc*analysis, the importance of bias field correction in the preprocessing was highlighted to mitigate inter-scanner variation in the GM volume estimation. Furthermore, the consistency of effect size for disease risk over scanners can benefit from image harmonization.

The SP-ComBat adapted the ComBat paradigm to model the inter-scanner variability in the image domain to evaluate the inconsistency of relative signal intensity and its uncertainty across scanners. In the ComBat equation, the location (γ) and scale (δ) terms are estimated to adjust the feature distribution during the harmonization process (Radua et al., 2020). The location term represents the shift in a particular feature’s distribution. By acquiring matched data from traveling subjects across scanners, we can assume that there were systemic shifts in signal intensity of the same tissue between cross-scanner data due to overall differences in scanner-related settings such as hardware and imaging protocols regardless of biological variation. The contrast differences can be inferred from the shift in relative signal intensity distributions given the use of global intensity normalization. On the other hand, the scale effect characterizes the variance of the feature distribution (Radua et al., 2020), reflecting the uncertainty of measurements. The novelty of SP-ComBat was to make ComBat modeling practical at the image level. While implementing VoxelComBat with subjects as observations may seem intuitive (Supplementary Material S2), it comes with certain limitations, including spatial inconsistency of the same voxel across subjects, potential insufficient sample sizes during model estimation, and an excessive number of the estimated parameters. Consequently, VoxelComBat may not be an ideal approach for assessing inter-scanner variability and harmonization performance. In contrast, when employing 3D superpixel parcellation to segment T1-weighted images into superpixels and considering each one as a feature with voxels as observations, the ComBat model can effectively approximate the location and scale of inter-scanner variability on an individual basis in the image domain. This approach offers a more robust and practical manner to estimate parameters, enhancing its feasibility for evaluating harmonization and investigating inter-scanner variation. Furthermore, the presented method holds the potential for application to other types of imaging modalities as long as the primary cause of the inter-scanner variability is contrast difference. While this framework relies on the availability of matched data, the increasing prevalence of large traveling subject cohorts serving as gold-standards for harmonization evaluation enables researchers to employ our method to validate the efficacy of newly developed harmonization methodologies (Hu et al., 2023;Marzi et al., 2024;Warrington et al., 2023).

**Fig. 11.**
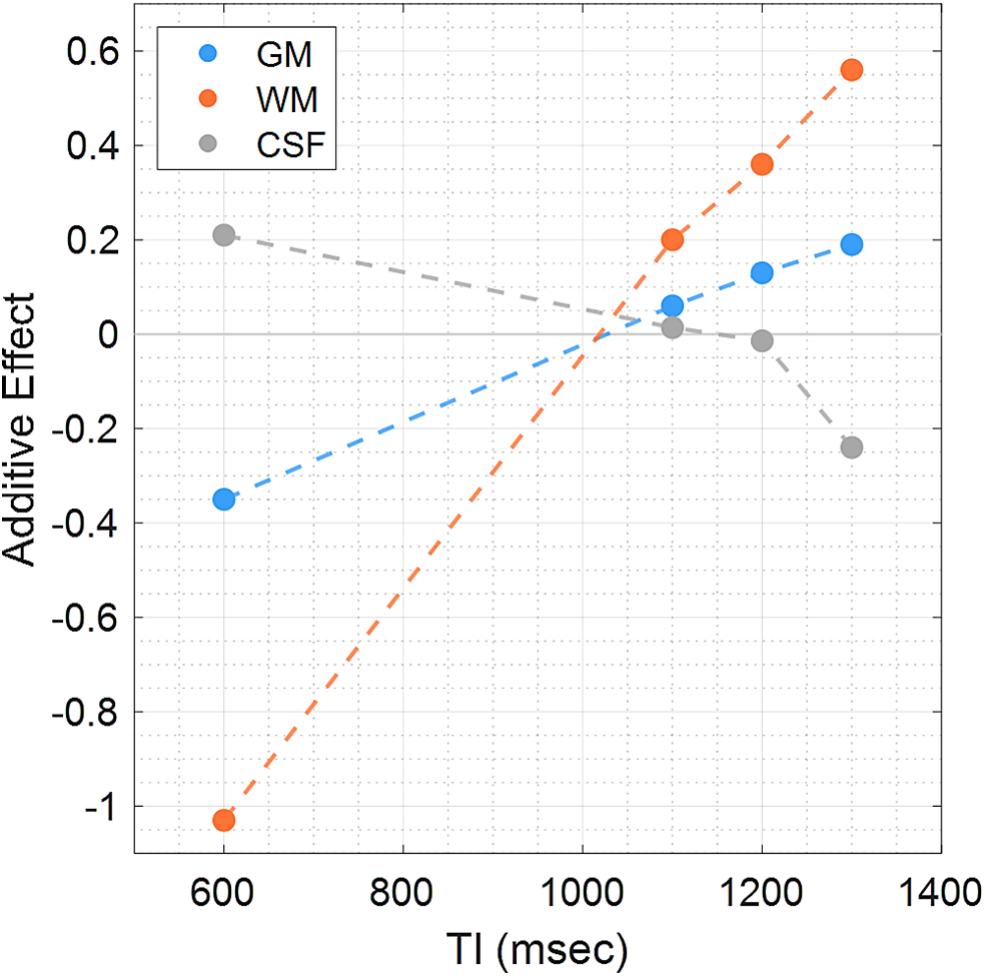
Scatter diagram illustrating the relationship between the imaging parameter, inversion time (TI), and the additive effects in major brain tissue classes. As TI increased, there was a tendency for positive deviations in the additive effects in gray matter (GM) and white matter (WM), while negative deviations were observed in cerebrospinal fluid (CSF), suggesting that the relative signal deviation, representing contrast differences across scanners, may be influenced by the setting of TI in T1-weighted imaging.

T1-weighted imaging holds significant prominence in clinical and research settings by offering important anatomical information of the brain (Bethlehem et al., 2022;Zhao et al., 2023). T1-weighted images with sufficient quality should exhibit clearly distinct tissue contrast between GM, WM, CSF, and pathology-related regions. This inherent contrast arises from differences in the longitudinal relaxation time (i.e., T1) among brain tissues. By adjusting various imaging parameters such as repetition time (TR), echo time (TE), and inversion time (TI), tissue contrasts in T1-weighted images can be optimized; typically, GM appears with intermediate signal intensity, while WM and CSF exhibit higher and lower signal intensity, respectively (Table 1) (Brown et al., 2014). MPRAGE and IR-FSPGR are the most common imaging sequences for T1-weighted imaging; the former is frequently implemented at Siemens and Philips scanners, whereas the latter is implemented at GE scanner (Jack Jr et al., 2010). Both sequences include an inversion recovery (IR) pulse design (Brown et al., 2014); the IR pulse selectively nulls the signal from certain tissues by manipulating TI, resulting in a range of T1-weighted contrasts and highlighting specific contrasts of interest. Hence, maintaining consistency of TI is crucial for effectively pooling cross-scanner T1-weighted images. The data used in this study were acquired with slightly different TI settings among scanners. We observed that the changes in TI would potentially affect the relative signal intensity in brain tissues across scanners, and these signal differences can be captured by the additive effects using SP-ComBat (Fig. 11). With an increase in TI, a trend towards positive deviations in the additive effects for GM and WM was observed, whereas a negative association was noticed in the CSF. This observation of signal deviation aligns with the empirical contrast mechanism in the physics of magnetic resonance (Brown et al., 2014); before the null point of CSF during the inversion recovery, longer TI would result in the reduced signal in CSF, the increased signal in GM and WM, and the diminished demarcation between GM and WM (as presented inTable 1, Philips exhibited the longest TI and the lowest GM/WM contrast compared to other scanners). The finding suggests that the setting of TI in T1-weighted imaging across scanners may have a substantial impact on the relative signal deviation of brain tissues, and this also confirms the effectiveness of using SP-ComBat to reflect inter-scanner variability. Nevertheless, as MPRAGE and IR-FSPGR employ distinct imaging acquisition schemes, the resultant overall image quality and contrast would also be influenced by other imaging parameters. Further investigation is warranted to comprehensively explore the impact of imaging parameters and sequences on tissue contrasts and image quality across scanners.

The ComBat harmonization ensures that the data from different scanners would share similar statistical properties given a normative distribution, making them directly comparable and suitable for joint analyses (Radua et al., 2020;Reynolds et al., 2023). In addition to establishing the normative range in terms of signal intensity, normative distributions of various image quality metrics should also be defined for the evaluation and comparison of image harmonization methodologies. We used an open-access package, MRIQC, to assess general image quality of T1-weighted images (Esteban et al., 2017,^2019^). The provided image quality reports can serve as a reference for future studies, offering comprehensive evaluations of image characteristics. Furthermore, one approach to enhance harmonization is by focusing on imaging preprocessing steps. As we demonstrated inFigure 9, proper image preprocessing, such as bias field correction, can effectively reduce inter-scanner variability and improve image qualities. However, we only assessed the impact of two main preprocessing steps (i.e., denoise and debias) in this study. Future investigations should encompass a broader range of image preprocessing techniques and quality assurance procedures to comprehensively evaluate their respective impacts on data harmonization and image quality. Besides, technical factors should also be considered to address inter-scanner variability; for example, main magnetic field strength (e.g., 1.5T, 3T, and 7T) (Han et al., 2006;Hu et al., 2023), magnetic field inhomogeneity (e.g., static field inhomogeneity, radiofrequency field non-uniformity, and gradient non-linearity) (Jovicich et al., 2006,^2009^), imaging protocols (e.g., imaging sequence, acquisition scheme, and parameters) (Hedges et al., 2022;Jovicich et al., 2009), image reconstruction (e.g., reconstruction method, sampling approach, filtering method, acceleration mode) (Deshmane et al., 2012;Hansen & Kellman, 2015), and more. Harmonization strategies may hold distinct advantages and disadvantages for biological signal detection. For example, although deep learning boosts effect sizes after harmonization, the consistency of biological estimates may suffer (Fig. 10). Different harmonization techniques offer various tradeoffs between precision and accuracy concerning imaging features. Further comparisons of these tradeoffs could better guide analytic choices based on applications. Nevertheless, under the unharmonized image condition, it appears to be a discernible common trend in the effect size profiles across scanners, which may suggest the potential for achieving data harmonization through meta- or mega-analysis techniques to consolidate statistics across sites and scanners (De Stefano et al., 2022;Radua et al., 2020). Given the growing use of machine-learning techniques in neuroimaging data analysis (C.-L. Chen, Hwang, et al., 2022;C.-L. Chen, Kuo,Chen, et al., 2022), harmonization procedures should also consider the subsequent analyses to avoid the data leakage issue that would potentially affect the downstream performance (Marzi et al., 2024). The SP-ComBat was designed to estimate the scanner-related parameters based on a held-out set for harmonization, which can help mitigate the influence of information leakage.

There are several limitations in this study that warrant consideration for future research. First, the overall sample size in the current study was relatively small due to the scarcity of traveling subjects across sites. The reported result should be interpreted cautiously when generalized to larger cohorts or samples potentially from other populations such as clinical patients. Second, the assumption of independence and identically distributed data (i.i.d.) within superpixels may be violated in certain cases, potentially influencing the parameter estimation. Addressing this limitation requires advanced statistical methods that account for dependent observations in this scenario. The current study did not investigate the impact of different imaging parameter settings and field strengths, which are known to influence image quality and tissue contrasts (Han et al., 2006;Jovicich et al., 2009). Moreover, although we found that the denoising step may not significantly contribute to the reduction of inter-scanner variation, noise levels can vary with different field strength, potentially introducing additional variability in the harmonization process. Furthermore, the reliance on superpixel parcellation in our pipeline may lead to partial volume effects, which could affect tissue class assignments. Exploring alternative parcellation methods may mitigate this limitation. In addition, the present study focused solely on GM, WM, and CSF tissues, which may overlook potential inter-scanner variability in other tissue types such as WM lesions. Additionally, the proposed framework was tested only on T1-weighted images, leaving its applicability to other imaging modalities, such as diffusion MRI and fMRI, unexplored in this study. However, the core component of our method (linear site effect removal using SP-ComBat-derived parameters) was designed to perform contrast adjustments across scanners, so the method can theoretically be generalized to other structural imaging sequences which are affected by inter-scanner contrast difference such as T2-weighted imaging. The current proposed pipeline was designed with the assumption that matched human phantoms would be available across scanners. Further investigation is warranted to test the feasibility of using pseudo-matched data from different sites (Karayumak et al., 2019). For the advancement of explainable harmonization (Munroe et al., 2024), a systematic analysis comparing statistical and deep learning harmonization methods is also warranted in future research.

In conclusion, by developing an analytic pipeline to characterize the location and scale effects of inter-scanner variability in T1-weighted images across scanners, we demonstrated the spatial patterns of relative signal deviation across scanners and leveraged the procedure to achieve interpretable image harmonization. Additionally, the ComBat-derived parameters can be used to quantify relative signal deviation and variation among scanners, enabling to assess harmonization performance. In the pursuit of robust harmonization methodologies for neuroimaging research, several crucial considerations (e.g., imaging acquisition, preprocessing, and modality-specific quality assessment) emerge to shape the future landscape of image harmonization. Understanding their influence on data variability is crucial to tailor effective strategies that accommodate these nuances. Although the harmonization strategy that we proposed mitigated approximately 40% of inter-scanner variation, the remaining 60% calls for advanced harmonization techniques. With collaborative efforts to incorporate advanced statistics and/or deep learning-based methods as well as hierarchical data processing strategies, newly developed harmonization methodologies are expected to unlock the potential to overcome complex inter-scanner variation, unveiling the intricacies of brain structure to facilitate scientific discoveries in the realm of neuroscience.

## Supplementary Material

Supplementary Material

## Data Availability

The data used in this study are not available due to the confidentiality agreement of the Research Ethics Committee. Scripts of analytic methods and codes of imaging process are available upon request from the corresponding author. The source code of the proposed SP-ComBat implemented with MATLAB can be found in our GitHub repository (https://github.com/ChangleChen/SPComBat).
