# Supplementary material for: Superpixel-ComBat modeling: A joint approach for harmonization and characterization of inter-scanner variability in T1-weighted images
Source: Imaging Neurosci (Camb). 2024 Oct 3;2:imag-2-00306. doi: 10.1162/imag_a_00306 (PMC12290534; doi:10.1162/imag_a_00306)
Supplement: Supplementary Material [file imag_a_00306-supp.pdf]

Supplementary Materials

S1 Specifics of superpixel size setting

S2 Comparison between VoxelComBat and SP-ComBat

S3 Evaluate harmonization performance using CNN-based scanner classification

S4 Tissue-based ComBat parameters representing site effects

S5 Tissue-based ComBat parameters after SP-ComBat harmonization

S6 Coefficients of variation of gray matter regional estimates after harmonization

S1 Specifics of superpixel size setting

In the image-driven 3D superpixel segmentation, a constraint was imposed to ensure sufficient number of voxels (a minimum of 27 voxels) contained in a superpixel for the subsequent ComBat modeling. The selection of the 27-voxel threshold was based on empirical observations derived from our experiments for practical considerations. Given that the image dimensions in the standard space were 121 × 145 × 121 voxels, the distribution of superpixel sizes resulting from the initial superpixel parcellation of a brain scan was analyzed. In general, the histograms of these distributions consistently showed a peak around the 27-voxel mark (Supplementary Figure 1), indicating that this size was frequently observed in the dataset. This threshold of 27 voxels was chosen since it aligns with accepted sample size requirements for statistical estimation, ensuring that each superpixel contains enough observations for robust parameter estimation during the ComBat modeling step. This size may also match the commonly used kernel size (3×3×3 voxels) for image operations such as convolution. Notably, different threshold values could affect the results; smaller superpixel sizes might increase the number of superpixels, leading to higher computational complexity and potential noise due to the inclusion of smaller, less representative regions. Conversely, larger superpixel sizes could result in a loss of spatial specificity, potentially oversimplifying the underlying tissue patterns. Therefore, the 27-voxel threshold represents an optimal balance given the image dimensions in the current study.

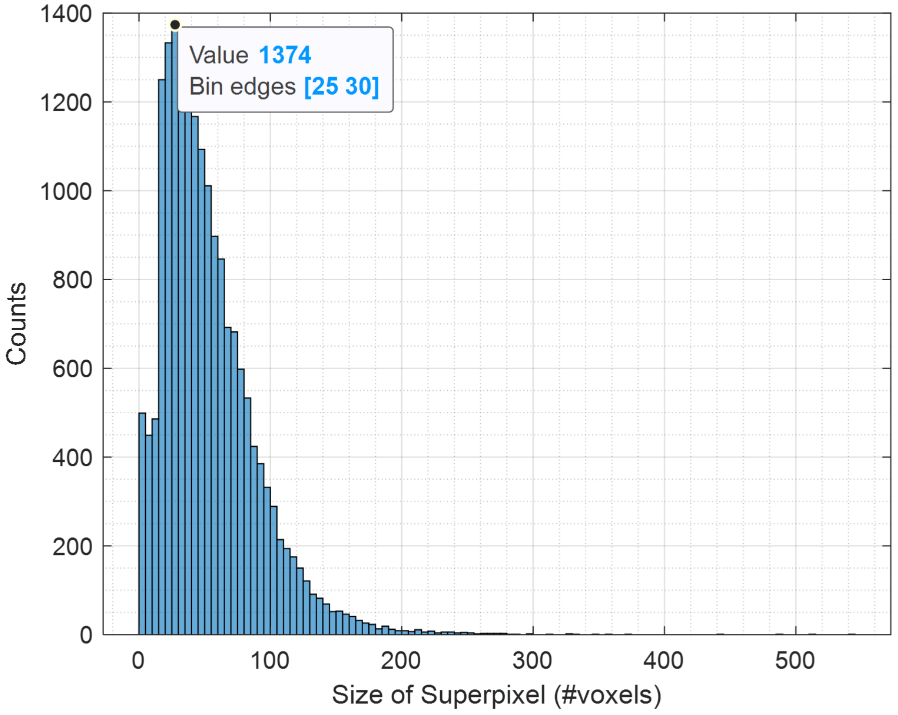

Supplementary Figure 1. Histogram of superpixel size in a brain scan. This histogram demonstrates the frequency of how many voxels contained in a superpixel after the initial superpixel segmentation.

S2 Comparison between VoxelComBat and SP-ComBat

ComBat is one of the most popular harmonization approach to unify feature distributions across sites and scanners. In ComBat modeling, image-derived measures such as cortical volume or thickness would be treated as features with subjects as observations. Thus, empirical Bayes estimation is utilized to estimate location and scale effects due to relatively small sample size in ComBat. Based on the typical intended use of ComBat, an intuitive way to directly implement ComBat model on images is to treat voxels as features and subjects as observations. Here, we implemented the voxel-wise ComBat (VoxelComBat) and use it to estimate parametric maps in the image domain.

The image preprocessing for VoxelComBat was the same as described in Method 2.2 including standard image preprocessing steps (e.g. image resize, image denoise, and bias correction) and spatial registration (i.e. two-step registration to the MNI space). The difference of VoxelComBat from the SP-ComBat was that, in the VoxelComBat, the ComBat model was directly applied to the voxels in the MNI space, estimating the additive and multiplicative terms for each voxel. As result, two parametric maps (i.e. additive and multiplicative terms) were obtained at the group level (Supplementary Figure 2). By visual inspection, the main contrasts of parametric maps derived from VoxelComBat were similar to those obtained by using SP-ComBat; for example, the additive terms in WM at GE were both lower than the average, and the multiplicative terms in WM at Philips were both higher than the normative range. However,

the parametric maps in VoxelComBat were noisier, especially in the multiplicative terms. We further performed quantitative analysis between VoxelComBat and SP-Combat approaches by estimating spatial correlation between parametric maps. Regarding the additive terms, the parametric maps derived from two approaches revealed high correlation at all scanners (GE: rho = 0.8671, Philips: rho = 0.8685, SiemensP: rho = 0.7663, SiemensT: rho = 0.7600, all *p*-values < 0.001), suggesting high similarity of the estimated location effects of inter-scanner variability between two ComBat implementations. On the other hand, regarding the multiplicative terms, the parametric maps obtained by two methods showed moderate correlation (GE: rho = 0.5202, Philips: rho = 0.6221, SiemensP: rho = 0.4521, SiemensT: rho = 0.3526, all p-values < 0.001), denoting relatively higher discrepancy of the estimated multiplicative terms between two implementations compared to the additive estimates.

However, using VoxelComBat implementation encounters a few limitations. First, because subjects were taken as observations, to estimate the parameters, the images had to be spatially registered to a common space. Due to inter-subject variability, the registered images may not perfectly align together, and this misalignment would further result in inaccurate estimation in ComBat. Also, the implementation would be affected by the sample size of traveling subjects. Compared to the SP-ComBat method, excess parameters in VoxelComBat had to be computed. Moreover, VoxelComBat is hard to be an individualized metric to evaluate inter-scanner variation and harmonization performance since the

estimation requires to be performed at the group level. These results may suggest that both implementations can yield similar estimated parameters at the group level, but the SP-ComBat is more flexible and practical than VoxelComBat. This confirms that the SP-ComBat method can effectively estimate the location and scale effects of inter-scanner variability.

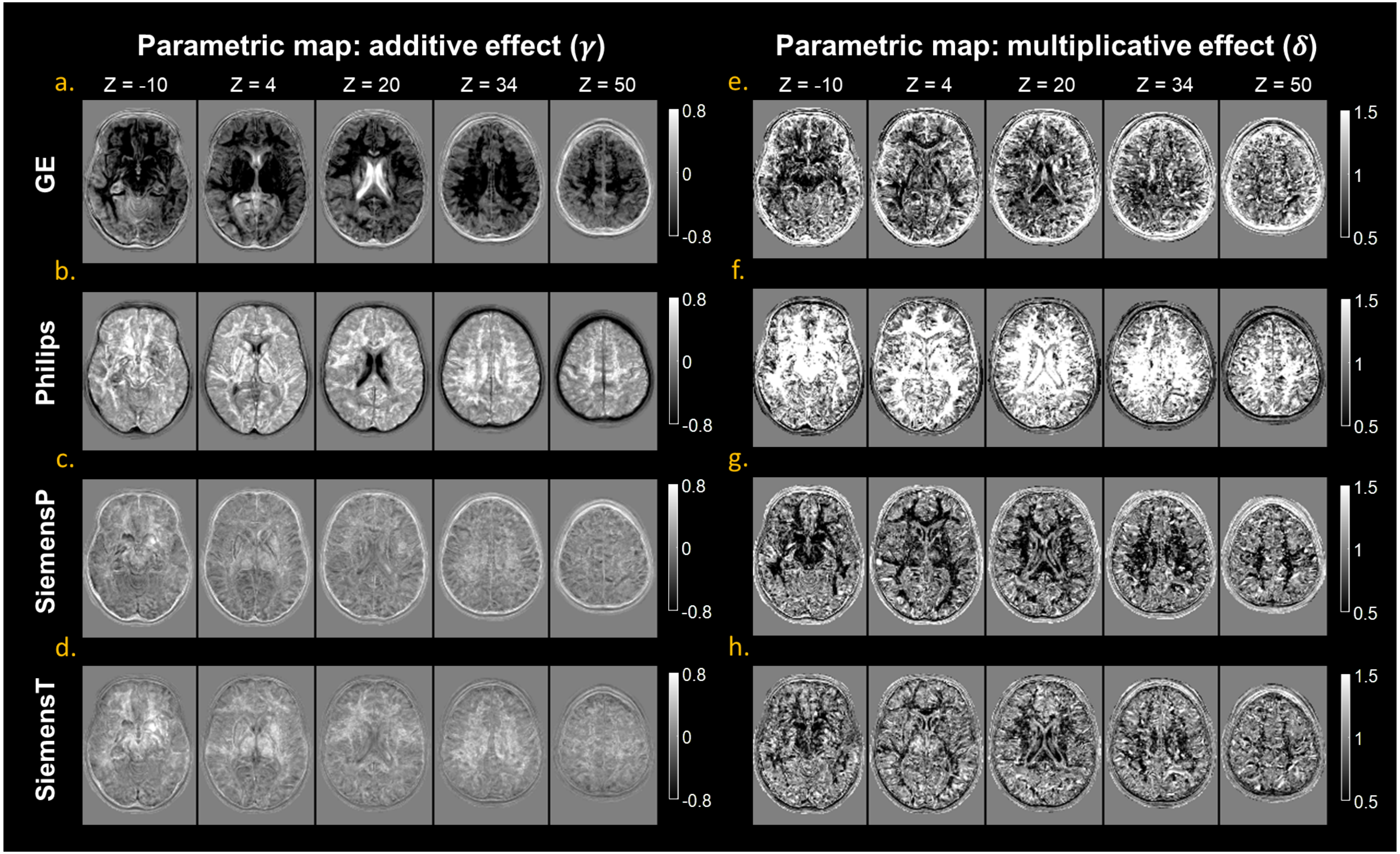

Supplementary Figure 2. Estimated maps demonstrating inter-scanner variability in the MNI space by using VoxelComBat. The parametric maps depict the additive term (γ) and multiplicative term (δ) specific to each scanner. The former represents the shift in signal distribution relative to the average (i.e. location effect) while the latter demonstrates the spread of the distribution (i.e. scale effect). The slices are sequentially

displayed from inferior (z = -10) to superior (z = 50) level in the brain.

S3 Evaluate harmonization performance using CNN-based scanner classification

In addition to conventional metrics for evaluating harmonization performance via harmonized images and their derivatives, we developed convolutional neural network (CNN) classifiers to predict scanner effects at the image level. This algorithm-agnostic, image-driven approach provided a more direct and robust assessment of harmonization methods. We hypothesized that the trained CNN classifiers would accurately identify scanner sources from raw T1-weighted images while their accuracy would significantly decrease when applied to successfully harmonized images.

We performed the experiment under five conditions including (1) raw (unharmonized), (2) CALAMITI, (3) MISPEL, (4) VoxelComBat, and (5) SPComBat images. To ensure fair comparisons across all conditions, all images were registered to the MNI space. In practice, subjects were randomly divided into training (N = 9) and test (N = 9) sets. Two-dimensional CNN classifiers were trained under each condition to predict the scanner source using a multiclass classification approach. The classifiers' input comprised 2D axial slices sampled from the central part of the brain volumes; specifically, we extracted 73 slices centered around the middle of the brain (± 36 slices from the center) from each scan, resulting in a total of 2,628 images (73 slices × 9 subjects × 4 scanners) for model training. The output was the scanner source label (GE, Philips, SiemensP, and SiemensT). The image classifiers utilized

an InceptionV3 pre-trained model as the base with additional layers including global average pooling, a dense layer with 1,024 neurons and ReLU activation, and a final dense layer with softmax activation for multiclass classification. The model was compiled with the Adam optimizer and sparse categorical crossentropy loss. The model training was conducted with a validation split (20% of the training samples) over 30 epochs with a batch size of 16. Early stopping was employed (tolerance of 5 epochs) to monitor validation loss and save the best model. This training process was repeated 10 times for each condition to estimate the standard deviation of model performance. The trained models were then applied to the test set to evaluate accuracy.

S4 Tissue-specific location (γ) and scale (δ) effects across scanners

	Additive effect (γ)			Multiplicative effect (δ)		
	GM	WM	CSF	GM	WM	CSF
GE	**-0.35 (0.20)*** **	**-1.03 (0.42)*** **	**0.21 (0.16)*** **	**1.04 (0.05)** **	0.99 (0.08)	**0.89 (0.06)*** **
Philips	0.19 (0.42)	**0.56 (1.03)* **	**-0.24 (0.21)*** **	**1.19 (0.08)*** **	**1.32 (0.12)*** **	**1.22 (0.13)*** **
SiemensP	0.06 (0.21)	0.20 (0.49)	0.01 (0.10)	**0.92 (0.04)*** **	**0.89 (0.08)*** **	1.00 (0.08)
SiemensT	**0.13 (0.19)* **	**0.36 (0.44)** **	-0.01 (0.15)	**0.92 (0.05)*** **	**0.89 (0.07)*** **	**0.97 (0.06)* **

Note: bold text indicates the comparison with significance: *: p-values < 0.05, **: p-values < 0.01, ***: p-values < 0.001.

Abbreviation: GM: gray matter, WM: white matter, CSF: cerebrospinal fluid.

S5 Tissue-specific location (γ) and scale (δ) effects across scanners after SP-Combat harmonization

	Additive effect (γ)			Multiplicative effect (δ)		
	GM	WM	CSF	GM	WM	CSF
GE	-0.02 (0.10)	0.03 (0.21)	-0.07 (0.08)**	1.00 (0.03)	0.99 (0.04)	1.09 (0.04)***
Philips	0.10 (0.11)**	0.08 (0.24)	0.17 (0.08)***	1.07 (0.03)***	1.02 (0.06)	1.05 (0.07)**
SiemensP	-0.05 (0.15)	-0.06 (0.30)	-0.03 (0.11)	0.99 (0.03)	1.01 (0.05)	0.96 (0.05)**
SiemensT	-0.02 (0.08)	-0.03 (0.18)	-0.05 (0.10)*	1.00 (0.03)	1.01 (0.05)	0.94 (0.05)***

Note: asterisk used to indicate the comparison with significance: *: *p*-values < 0.05, **: *p*-values < 0.01, ***: *p*-values < 0.001.

Abbreviation: GM: gray matter, WM: white matter, CSF: cerebrospinal fluid.

S6 Coefficients of variation of gray matter regional estimates after harmonization

ROI	Raw	CALAMITI	MISPEL	VoxelComBat	SPComBat
lSupFroG	0.0444 (0.0252)	0.0504 (0.0186)<br>pval = 0.442	0.0403 (0.0108)<br>pval = 0.4582	**0.0232 (0.0127)<br>pval = 0.0025**	**0.0228 (0.0147)<br>pval = 0.0068**
rSupFroG	0.0441 (0.019)	0.0527 (0.0187)<br>pval = 0.1891	0.0341 (0.0158)<br>pval = 0.1331	**0.0241 (0.0131)<br>pval = 0.0001**	**0.0259 (0.0073)<br>pval = 0.0007**
lMidFroG	0.0467 (0.0183)	0.0577 (0.0245)<br>pval = 0.1131	0.0408 (0.0188)<br>pval = 0.448	**0.0281 (0.0173)<br>pval = 0.005**	**0.0203 (0.0112)<br>pval < 0.0001**
rMidFroG	0.044 (0.0199)	0.0485 (0.0223)<br>pval = 0.4822	0.0463 (0.0196)<br>pval = 0.7888	**0.0219 (0.011)<br>pval = 0.0001**	**0.0170 (0.013)<br>pval < 0.0001**
lInfFroG	0.0368 (0.0148)	0.0526 (0.0205)<br>pval = 0.0036	0.0356 (0.0134)<br>pval = 0.7962	0.0288 (0.0111)<br>pval = 0.1104	**0.0203 (0.0093)<br>pval = 0.0009**
rInfFroG	0.0338 (0.017)	0.0489 (0.0176)<br>pval = 0.0111	0.0372 (0.0189)<br>pval = 0.6122	0.0224 (0.0117)<br>pval = 0.0231	**0.0208 (0.0125)<br>pval = 0.0275**
lPrcG	0.0590 (0.0302)	0.0839 (0.0301)<br>pval = 0.0434	0.0558 (0.0179)<br>pval = 0.6868	0.0508 (0.0243)<br>pval = 0.2592	0.0681 (0.0299)<br>pval = 0.1811
rPrcG	0.0654 (0.0336)	0.0863 (0.0341)<br>pval = 0.0729	0.0526 (0.0234)<br>pval = 0.1534	**0.0415 (0.0155)<br>pval = 0.0062**	0.0659 (0.0273)<br>pval = 0.9252
lMidOrbG	0.0434 (0.0166)	0.0710 (0.0411)<br>pval = 0.0378	0.0517 (0.0286)<br>pval = 0.1983	0.0426 (0.0134)<br>pval = 0.9112	**0.0231 (0.0109)<br>pval = 0.0003**
rMidOrbG	0.0404 (0.0203)	0.0754 (0.0344)<br>pval = 0.007	0.0478 (0.0173)<br>pval = 0.2377	0.0343 (0.0142)<br>pval = 0.362	**0.0195 (0.0095)<br>pval = 0.0007**
lLatOrbG	0.0559 (0.0261)	0.0622 (0.0282)<br>pval = 0.4224	0.0502 (0.0284)<br>pval = 0.2234	**0.0294 (0.0152)<br>pval = 0.0032**	**0.0236 (0.0196)<br>pval = 0.0036**
rLatOrbG	0.0765 (0.0406)	0.065 (0.0243)<br>pval = 0.367	**0.0520 (0.027)<br>pval = 0.0081**	**0.0313 (0.0145)<br>pval = 0.0022**	**0.0244 (0.0156)<br>pval = 0.0002**
lRecG	0.0513 (0.0221)	0.0657 (0.029)<br>pval = 0.174	**0.0397 (0.0218)<br>pval = 0.04**	0.0397 (0.0226)<br>pval = 0.1446	**0.0332 (0.0168)<br>pval = 0.0289**
rRecG	0.0586 (0.03)	0.0549 (0.0206)<br>pval = 0.6776	0.0473 (0.0252)<br>pval = 0.201	**0.0380 (0.0213)<br>pval = 0.0016**	**0.0277 (0.014)<br>pval = 0.0001**
lPoCG	0.0573 (0.0229)	0.0800 (0.0336)<br>pval = 0.1022	0.0522 (0.0157)<br>pval = 0.5325	**0.0325 (0.0224)<br>pval = 0.003**	0.0508 (0.0328)<br>pval = 0.4506
rPoCG	0.0737 (0.0343)	0.0840 (0.0271)<br>pval = 0.4134	**0.0468 (0.0104)<br>pval = 0.0116**	**0.0318 (0.0106)<br>pval = 0.0002**	**0.0530 (0.0226)<br>pval = 0.0181**
lSupParG	0.0613 (0.0384)	0.0773 (0.0195)<br>pval = 0.1878	0.0502 (0.0181)<br>pval = 0.2166	0.0442 (0.0131)<br>pval = 0.1166	**0.0344 (0.0181)<br>pval = 0.0031**

rSupParG	0.0695 (0.0336)	0.0783 (0.0279) pval = 0.4819	**0.0378 (0.0157) pval = 0.0009**	**0.0358 (0.0175) pval = 0.0015**	**0.0310 (0.017) pval = 0.0014**
lSupMarG	0.0327 (0.0166)	0.0608 (0.0289) pval = 0.0025	0.0370 (0.0138) pval = 0.3437	0.0514 (0.0194) pval = 0.0268	0.0350 (0.0209) pval = 0.7552
rSupMarG	0.0450 (0.0252)	0.0813 (0.0311) pval = 0.0036	0.0541 (0.0258) pval = 0.3894	0.0313 (0.0133) pval = 0.088	**0.0280 (0.0201) pval = 0.033**
lAngG	0.0462 (0.0346)	0.0560 (0.0277) pval = 0.3983	0.0396 (0.0136) pval = 0.5144	0.0496 (0.0151) pval = 0.773	0.0368 (0.0198) pval = 0.386
rAngG	0.0558 (0.0289)	0.0837 (0.0302) pval = 0.0147	0.0398 (0.0157) pval = 0.0606	0.0408 (0.0143) pval = 0.098	**0.0293 (0.0118) pval = 0.0044**
lPCu	0.0667 (0.0254)	0.0716 (0.0209) pval = 0.5095	**0.0499 (0.0146) pval = 0.0094**	**0.0247 (0.0123) pval < 0.0001**	**0.0260 (0.0127) pval < 0.0001**
rPCu	0.0657 (0.0206)	0.0632 (0.0169) pval = 0.7346	**0.0381 (0.0186) pval = 0.0021**	**0.0234 (0.0147) pval < 0.0001**	**0.0248 (0.0096) pval < 0.0001**
lSupOccG	0.0858 (0.0309)	0.0804 (0.0393) pval = 0.696	**0.0490 (0.0287) pval = 0.0013**	**0.0342 (0.0145) pval < 0.0001**	**0.0492 (0.0313) pval = 0.0048**
rSupOccG	0.0962 (0.029)	0.0613 (0.0245) pval = 0.0006	**0.0439 (0.0225) pval = 0.0001**	**0.0387 (0.0089) pval < 0.0001**	**0.0393 (0.0193) pval < 0.0001**
lMidOccG	0.0692 (0.0379)	0.0562 (0.0248) pval = 0.2447	**0.0385 (0.0096) pval = 0.0066**	0.0564 (0.0175) pval = 0.257	**0.0417 (0.0244) pval = 0.0059**
rMidOccG	0.0789 (0.0373)	0.0608 (0.0232) pval = 0.0706	**0.0347 (0.0139) pval = 0.0002**	**0.0275 (0.0092) pval = 0.0002**	**0.0287 (0.0168) pval = 0.0001**
lInfOccG	0.0539 (0.0338)	0.0753 (0.0336) pval = 0.024	0.0565 (0.0205) pval = 0.729	0.0387 (0.0184) pval = 0.0507	0.0416 (0.0236) pval = 0.1158
rInfOccG	0.0611 (0.0261)	0.0718 (0.023) pval = 0.214	**0.0389 (0.0231) pval = 0.0075**	0.0400 (0.0283) pval = 0.0574	**0.0359 (0.0211) pval = 0.0058**
lCun	0.1147 (0.0356)	0.0956 (0.0249) pval = 0.0924	**0.0783 (0.0489) pval = 0.0078**	**0.0449 (0.0331) pval = 0.0001**	**0.0477 (0.0257) pval < 0.0001**
rCun	0.0836 (0.0387)	0.0779 (0.03) pval = 0.6271	0.0774 (0.0481) pval = 0.7015	**0.0514 (0.0236) pval = 0.0083**	**0.0488 (0.025) pval = 0.0132**
lSupTemG	0.0321 (0.0116)	0.0514 (0.0228) pval = 0.0076	0.0326 (0.0135) pval = 0.8782	**0.0221 (0.0054) pval = 0.0141**	**0.0147 (0.0082) pval = 0.0001**
rSupTemG	0.0367 (0.0198)	0.0611 (0.0247) pval = 0.0096	0.0347 (0.0116) pval = 0.7136	**0.0198 (0.012) pval = 0.0206**	**0.0149 (0.0076) pval = 0.0007**
lMidTemG	0.0166 (0.0053)	0.0542 (0.0285) pval = 0.0001	0.0275 (0.0129) pval = 0.0105	0.0282 (0.0093) pval = 0.0012	0.0195 (0.0088) pval = 0.2893
rMidTemG	0.0240 (0.0165)	0.0594 (0.0268) pval = 0.0001	0.0394 (0.0195) pval = 0.0289	0.0259 (0.0098) pval = 0.628	0.0216 (0.0094) pval = 0.5719
lInfTemG	0.0250 (0.0128)	0.0663 (0.0168) pval < 0.0001	0.0364 (0.0146) pval = 0.0164	0.0263 (0.0146) pval = 0.8106	0.0240 (0.0091) pval = 0.8072

rInfTemG	0.0342 (0.0132)	0.0675 (0.029)<br>pval = 0.0004	0.0309 (0.017)<br>pval = 0.5514	0.0265 (0.0135)<br>pval = 0.2089	0.0284 (0.013)<br>pval = 0.1722
lParHipG	0.0491 (0.0219)	0.0507 (0.0185)<br>pval = 0.783	0.0422 (0.0195)<br>pval = 0.2514	**0.0359 (0.0148)**<br>**pval = 0.0251**	**0.0309 (0.0117)**<br>**pval = 0.0005**
rParHipG	0.0548 (0.0214)	0.0498 (0.0258)<br>pval = 0.4393	**0.0394 (0.0195)**<br>**pval = 0.0327**	**0.0359 (0.0222)**<br>**pval = 0.0029**	**0.0398 (0.0161)**<br>**pval = 0.0154**
lLinG	0.0884 (0.0194)	**0.0509 (0.024)**<br>**pval = 0.0001**	**0.0500 (0.0299)**<br>**pval < 0.0001**	**0.0343 (0.0216)**<br>**pval < 0.0001**	**0.0360 (0.0188)**<br>**pval < 0.0001**
rLinG	0.0765 (0.0253)	**0.0533 (0.029)**<br>**pval = 0.0372**	**0.0410 (0.0268)**<br>**pval = 0.0002**	**0.0354 (0.0184)**<br>**pval < 0.0001**	**0.0336 (0.0195)**<br>**pval < 0.0001**
lFusG	0.0412 (0.0141)	0.0689 (0.0239)<br>pval = 0.0002	0.0400 (0.0167)<br>pval = 0.6597	**0.0231 (0.0147)**<br>**pval = 0.0011**	**0.0250 (0.0101)**<br>**pval = 0.001**
rFusG	0.0435 (0.018)	0.0638 (0.0227)<br>pval = 0.0049	0.0357 (0.0136)<br>pval = 0.0705	**0.0265 (0.0125)**<br>**pval = 0.0031**	**0.0261 (0.0131)**<br>**pval = 0.0003**
lIns	0.0307 (0.0131)	0.0510 (0.0172)<br>pval = 0.0095	0.0423 (0.0162)<br>pval = 0.0315	0.0397 (0.0145)<br>pval = 0.1173	0.0273 (0.0104)<br>pval = 0.421
rIns	0.0311 (0.0146)	0.0536 (0.0237)<br>pval = 0.0154	0.0352 (0.0152)<br>pval = 0.4914	0.0284 (0.0119)<br>pval = 0.3805	**0.0226 (0.01)**<br>**pval = 0.037**
lCinG	0.0447 (0.0178)	0.0492 (0.0175)<br>pval = 0.459	**0.0342 (0.0116)**<br>**pval = 0.0305**	**0.0266 (0.0155)**<br>**pval = 0.0002**	**0.0217 (0.01)**<br>**pval < 0.0001**
rCinG	0.0392 (0.0152)	0.0538 (0.0271)<br>pval = 0.0509	0.0303 (0.013)<br>pval = 0.1094	**0.0249 (0.0121)**<br>**pval = 0.0012**	**0.0232 (0.0098)**<br>**pval = 0.0002**
lCau	0.1166 (0.0507)	**0.0657 (0.0339)**<br>**pval = 0.0016**	**0.0458 (0.0198)**<br>**pval = 0.0001**	**0.0299 (0.0243)**<br>**pval < 0.0001**	**0.0303 (0.0169)**<br>**pval < 0.0001**
rCau	0.1673 (0.0924)	**0.0666 (0.0249)**<br>**pval = 0.0008**	**0.0512 (0.0263)**<br>**pval < 0.0001**	**0.0319 (0.0212)**<br>**pval < 0.0001**	**0.0441 (0.0273)**<br>**pval < 0.0001**
lPut	0.1068 (0.0531)	0.1055 (0.0675)<br>pval = 0.9361	0.0981 (0.0408)<br>pval = 0.4324	**0.0416 (0.0185)**<br>**pval = 0.0001**	**0.0301 (0.0218)**<br>**pval < 0.0001**
rPut	0.1278 (0.0392)	0.1254 (0.0552)<br>pval = 0.8931	0.1314 (0.0404)<br>pval = 0.7292	**0.0558 (0.0248)**<br>**pval < 0.0001**	**0.0561 (0.0196)**<br>**pval < 0.0001**
lHip	0.0509 (0.0188)	0.0730 (0.0397)<br>pval = 0.0376	0.0608 (0.0228)<br>pval = 0.1447	**0.0343 (0.0164)**<br>**pval = 0.0063**	**0.0187 (0.0091)**<br>**pval < 0.0001**
rHip	0.0510 (0.0175)	0.0769 (0.0416)<br>pval = 0.0432	0.0580 (0.0318)<br>pval = 0.4097	0.0425 (0.0197)<br>pval = 0.1252	**0.0284 (0.0117)**<br>**pval = 0.0002**
bCBeL	0.0551 (0.0276)	0.0813 (0.0283)<br>pval = 0.0018	0.0461 (0.0267)<br>pval = 0.264	0.0408 (0.0207)<br>pval = 0.095	**0.0354 (0.0165)**<br>**pval = 0.0055**
bBst	0.3392 (0.1312)	**0.1700 (0.054)**<br>**pval = 0.0007**	0.3577 (0.0616)<br>pval = 0.5597	**0.1356 (0.0572)**<br>**pval = 0.0001**	**0.1513 (0.0626)**<br>**pval = 0.0001**

Note: values in bold indicate significantly *lower* coefficients of variation (CV) of gray matter regions of interest (ROI) compared to the raw (i.e. unharmonized) condition.

Abbreviation: lSupFroG, L superior frontal gyrus; rSupFroG, R superior frontal gyrus; lMidFroG, L middle frontal gyrus; rMidFroG, R middle frontal gyrus; lInfFroG, L inferior frontal gyrus; rInfFroG, R inferior frontal gyrus; lPrcG, L precentral gyrus; rPrcG, R precentral gyrus; lMidOrbG, L middle orbitofrontal gyrus; rMidOrbG, R middle orbitofrontal gyrus; lLatOrbG, L lateral orbitofrontal gyrus; rLatOrbG, R lateral orbitofrontal gyrus; lRecG, L gyrus rectus; rRecG, R gyrus rectus; lPoCG, L postcentral gyrus; rPoCG, R postcentral gyrus; lSupParG, L superior parietal gyrus; rSupParG, R superior parietal gyrus; lSupMarG, L supramarginal gyrus; rSupMarG, R supramarginal gyrus; lAngG, L angular gyrus; rAngG, R angular gyrus; lPCu, L precuneus; rPCu, R precuneus; lSupOccG, L superior occipital gyrus; rSupOccG, R superior occipital gyrus; lMidOccG, L middle occipital gyrus; rMidOccG, R middle occipital gyrus; lInfOccG, L inferior occipital gyrus; rInfOccG, R inferior occipital gyrus; lCun, L cuneus; rCun, R cuneus; lSupTemG, L superior temporal gyrus; rSupTemG, R superior temporal gyrus; lMidTemG, L middle temporal gyrus; rMidTemG, R middle temporal gyrus; lInfTemG, L inferior temporal gyrus; rInfTemG, R inferior temporal gyrus; lParHipG, L parahippocampal gyrus; rParHipG, R parahippocampal gyrus; lLinG, L lingual gyrus; rLinG, R lingual gyrus; lFusG, L fusiform gyrus; rFusG, R fusiform gyrus; lIns, L insular cortex; rIns, R insular cortex; lCinG, L cingulate gyrus; rCinG, R cingulate gyrus; lCau, L caudate; rCau, R caudate; lPut, L putamen; rPut, R putamen; lHip, L hippocampus; rHip, R hippocampus; bCBeL, bilateral cerebellum; bBst, bilateral brainstem.
